# Delivery Model Matters for Digital Interventions in Pain Self-Management: Mixed Methods Study

**DOI:** 10.2196/89780

**Published:** 2026-07-20

**Authors:** Lise Solberg Nes, Elin Børøsund, Elise Flakk Nordang, Rikke Aune Asbjørnsen, Hilde Eide, Cecilie Varsi, Lori B Waxenberg, Karen E Weiss, Eleshia J Morrison, Hanne Stavenes Støle, Christine Marie Rygg Hoksnes, Ólöf B Kristjansdottir, Milada Hagen, Audun Stubhaug, Karlein MG Schreurs

**Affiliations:** 1 Department of Digital Health Research Division of Medicine Oslo University Hospital Oslo Norway; 2 Institute of Clinical Medicine Faculty of Medicine University of Oslo Oslo Norway; 3 Department of Psychiatry and Psychology College of Medicine and Science Mayo Clinic Rochester, MN United States; 4 Department of Nursing and Health Sciences Faculty of Health and Social Sciences University of South-Eastern Norway Drammen Norway; 5 Department of Research and Innovation Vestfold Hospital Trust Tønsberg Norway; 6 Department of Psychology Health and Technology University of Twente Enschede The Netherlands; 7 Centre for Health and Technology Faculty of Health and Social Sciences University of South-Eastern Norway Drammen Norway; 8 Faculty of Health and Social Sciences University of South-Eastern Norway Drammen Norway; 9 Department of Clinical & Health Psychology College of Public Health and Health Professions University of Florida Gainesville, FL United States; 10 Department of Child Health and Development Norwegian Institute of Public Health Oslo Norway; 11 Mental Health Team West Primary Care of the Capital Area Reykjavik Iceland; 12 Faculty of Health Sciences Oslo Metropolitan University Oslo Norway; 13 Department of Pain Management and Research Oslo University Hospital Oslo Norway; 14 Regional Advisory Unit on Pain Oslo University Hospital Oslo Norway

**Keywords:** acceptance and commitment therapy, chronic pain, cognitive behavioral therapy, delivery models, digital health, efficacy, self-management

## Abstract

**Background:**

Digital health interventions can improve access to and outreach of evidence-based support for people living with chronic pain. How best to deliver such interventions remains unclear, however, and although guided or blended care models show promise, digital interventions are primarily delivered without support or follow-up.

**Objective:**

This study aimed to compare the use and effect of the evidence-based digital pain self-management intervention EPIO when (1) delivered through digital download without follow-up (ie, EPIO_NoFollowUp_), compared with (2) delivery through a simple blended-care model (ie, EPIO_BlendedCare_), or care-as-usual controls.

**Methods:**

People (n=73) with chronic pain received access to the EPIO program through individual download (EPIO_NoFollowUp_). Program use and outcome measures were collected over 12 months and compared with findings from a separate randomized controlled trial in which people with chronic pain (n=266) were assigned to receive EPIO through a simple blended care model (EPIO_BlendedCare_) or to a care-as-usual control group. Outcome measures were collected at baseline and at 3, 6, and 12 months and included pain interference (Brief Pain Inventory [BPI]; primary outcome), pain severity (BPI), anxiety and depression (Hospital Anxiety and Depression Scale), self-regulatory fatigue (Self-Regulatory Fatigue Scale-18), health-related quality of life (HRQoL; RAND 36-Item Health Survey), pain catastrophizing (Pain Catastrophizing Scale), and pain acceptance (Chronic Pain Acceptance Questionnaire-8). Generalized linear models for repeated measures were fitted to explore between-group differences over time. Interviews with the EPIO_NoFollowUp_ group (n=15) exploring perceived program experiences were analyzed using rapid analysis.

**Results:**

Participants (N=332) had a median age of 49 (IQR 39-55) years, were primarily women (271/332, 82%), and had a variety of pain conditions. Statistically significant between-group differences in favor of the EPIO_NoFollowUp_ group compared with the control group included improved HRQoL and pain acceptance and reduced pain catastrophizing. Intervention group comparison revealed only 1 significant between-group difference, for self-regulation, in favor of EPIO_BlendedCare_. Participants receiving the EPIO_NoFollowUp_ delivery model displayed lower program use, averaging 10 (IQR 3-24) days of use vs 30 (IQR 13-44) days for EPIO_BlendedCare_. Similarly, 23% (17/73) of the EPIO_NoFollowUp_ group participants completed ≥7 of 9 modules, compared with 62% (78/125) in EPIO_BlendedCare_. Higher education level was associated with significant improvements in pain severity, anxiety, HRQoL, and pain catastrophizing, as well as higher module completion, in the EPIO_NoFollowUp_ group but not in the EPIO_BlendedCare_ group. Qualitative findings identified a preference for more follow-up, as well as positive changes in awareness, acceptance, and coping in the EPIO_NoFollowUp_ group.

**Conclusions:**

The delivery model should be considered when providing access to digital self-management, and models with guidance appear to be the most helpful. When health care resources are limited, blended care delivery should likely be chosen for those who need it most, for example, those with lower education.

## Introduction

Chronic pain is disabling and entails a variety of individual and public health challenges [1-3]. For people living with chronic pain, physical and psychological health and well-being are often affected, subsequently affecting quality of life (QoL) as well as the capacity for self-regulation [1,4-6].

Recognizing the complex biopsychosocial aspects of pain [4], treatment approaches, including self-management interventions such as cognitive behavioral therapy (CBT [7]) and the related acceptance and commitment therapy (ACT [8]), have been recommended and shown to be beneficial [9-12]. These usually in-person self-management interventions, however, are not always offered or available [9], pointing to the potential of digital pain self-management programs to improve intervention access and outreach [13-15].

The current research team consequently designed and developed EPIO (ie, inspired by the Greek goddess for the soothing of pain, Epione), a digital self-management intervention for people living with chronic pain [16]. While digital health interventions show significant promise, a number of challenges have been identified, including limited or a lack of evidence-based content [17,18], limited or no stakeholder involvement during development [19], challenges with adherence and attrition [20,21], inadequate proof of short- and long-term efficacy [13,15,22,23], and limited, if any, evidence of implementation into practice [15,24].

The design and development of the EPIO program was consequently informed by evidence, with pervasive end user and health care provider input throughout and the inclusion of personalization aspects to support engagement and adherence [16,25,26]. Following recommendations for the development of complex interventions [27], EPIO was then tested in a feasibility pilot study, with people living with chronic pain describing EPIO as easy to use and reporting excellent user satisfaction [28]. Interviews conducted after the feasibility pilot study identified EPIO as raising awareness, fostering joy and enthusiasm, being a friend, and helping participants make peace with the pain [29]. In an ensuing randomized controlled trial (RCT) evaluating efficacy, people with chronic pain who had access to EPIO, compared with care-as-usual controls, reported reduced symptoms of depression and self-regulatory fatigue at 3 months [30], and reduced symptoms of anxiety and depression, reduced self-regulatory fatigue and pain catastrophizing, and improved health-related quality of life (HRQoL) at 12 months [31]. Post-RCT interviews revealed positive experiences based on access to and use of EPIO, including beneficial changes in cognition, coping, and engagement [32].

As guided digital interventions may have a greater impact than unguided interventions [33-35], EPIO has so far been delivered in a simple blended care delivery model (ie, with 1 face-to-face introduction session and 2 follow-up telephone calls [28-31]). Even such a simple form of blended care delivery requires resources, however, and given current strained health care systems and the predicted shortfall in health care providers [36], identifying ways to deliver pain self-management interventions in effective yet low resource-demanding ways [15] is hence vital.

In real-world settings, digital apps are usually downloaded individually, without additional support or follow-up, which underlines the need to explore the real-world utility of digital pain self-management interventions [15]. Enrolling people living with chronic pain, the current study therefore aimed to test the effectiveness and use of EPIO through:

- A 12-month, 1-arm trial using an individual download with a brief-information-only delivery model (ie, EPIO_NoFollowUp_), comparing this group with:
- A 12-month RCT intervention group with a simple blended care delivery model (ie, EPIO_BlendedCare_ [30,31]), and
- A 12-month RCT care-as-usual control group [30,31].

The goal was to explore whether program access through download with brief information only (ie, EPIO_NoFollowUp_) would be associated with significant improvements in primary (ie, pain interference) and secondary (ie, pain severity, depression, anxiety, self-regulatory fatigue, HRQoL, pain catastrophizing, and pain acceptance) outcomes over the course of 12 months. Participants receiving the intervention through EPIO_NoFollowUp_ were hypothesized to experience significant improvements in outcome measures compared with the care-as-usual control group, but similar or less improvement compared with those in the EPIO_BlendedCare_ group. Program use (eg, program progress and frequency of use) was examined on an exploratory basis, with lower program use predicted for the EPIO_NoFollowUp_ group compared with the EPIO_BlendedCare_ group. In addition, as education level might impact health intervention outcomes, the study sought to explore whether education level or related factors [37-39] would affect study outcomes.

## Methods

### Study Design

A quasi-experimental mixed methods design was used, with a single-arm group being compared with data from a separate RCT [30,31]. The single-arm group received access to the EPIO program with brief information upon download only (ie, EPIO_NoFollowUp_). Outcome measures and program use data were collected over 12 months, and findings were then compared with data from a previously conducted RCT [30,31], in which participants had either received EPIO through a simple blended care delivery model (EPIO_BlendedCare_) or had been randomized to a care-as-usual control group. Recruitment for the single-arm EPIO_NoFollowUp_ group took place after RCT recruitment completion (ie, the 3 groups were not enrolled simultaneously).

Participants in the study were people living with chronic pain for 3 months or more. Participants in the EPIO_NoFollowUp_ group were recruited between January and May 2022, whereas participants in the EPIO_BlendedCare_ and care-as-usual control groups (ie, RCT [31]) were recruited between November 2019 and February 2021. Inclusion criteria included (1) living with chronic pain for ≥3 months, (2) being aged ≥18 years, (3) having access to a smartphone or tablet, and (4) being able to understand and read Norwegian. In addition, participants in the RCT had to be able to attend a face-to-face introduction session. Exclusion criteria included migraine or cancer-related pain, as well as untreated severe psychological illness (ie, all self-reported).

### Study Procedure

#### Overview

**Figure 1:**
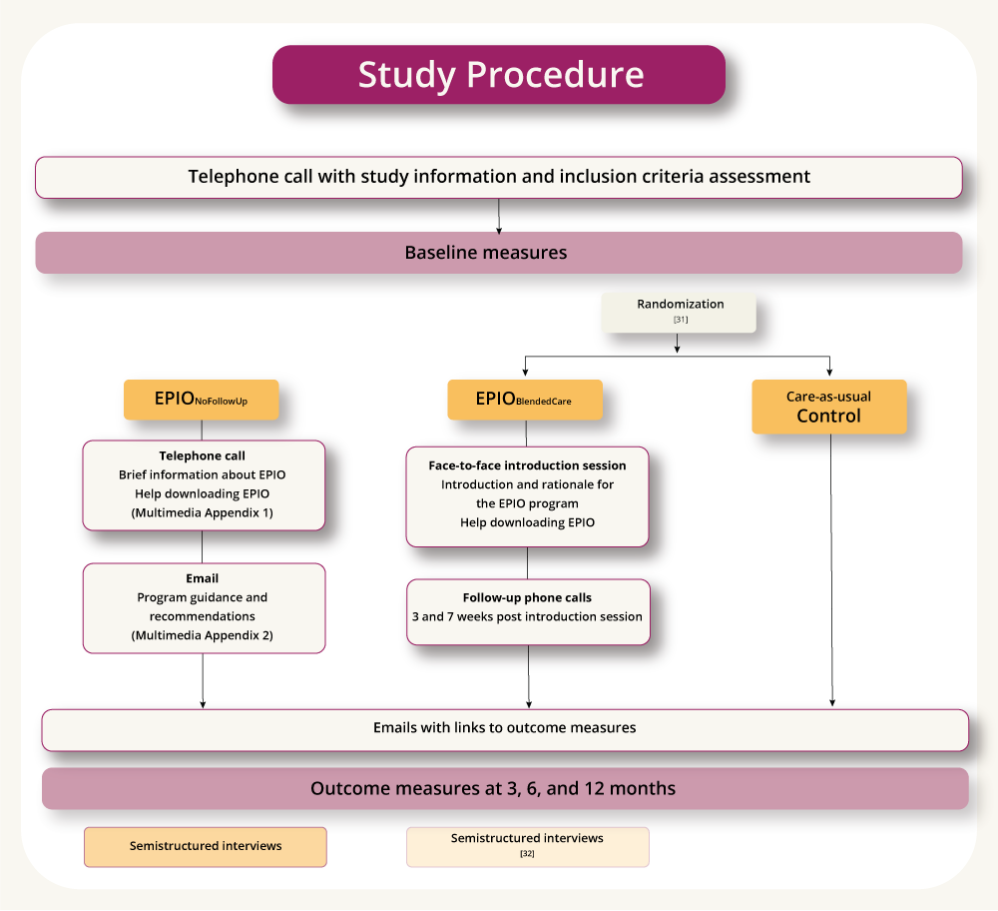
Overview of study procedures.

Potential participants (ie, all groups) were informed about the study through collaborating partners, via flyers distributed at various health care facilities (eg, a university hospital, local health care services, and primary care practices), or via social media or patient organizations’ web pages. Interested patients had their contact information forwarded to the project team or could contact the team via a study telephone number or website. A project team member subsequently provided detailed information about study participation. As the EPIO_NoFollowUp_ group was included after completion of recruitment for the RCT (ie, EPIO_BlendedCare_ and care-as-usual control groups), participants in the RCT were not informed about the EPIO_NoFollowUp_ group. Similarly, participants in the EPIO_NoFollowUp_ group were not specifically informed about the RCT groups. Information about the then-ongoing RCT (ie, study goals, recruitment, and randomization procedures) could nevertheless be obtained through online study pages by searching for such information. All included participants provided written informed consent before completing baseline outcome measures through a secure digital research server at Services for Sensitive Data (TSD; University of Oslo). Figure 1 [31,32] provides an overview of the study procedures.

#### EPIO_NoFollowUp_ Group

Participants in the single-arm EPIO_NoFollowUp_ group received a telephone call with brief information about the EPIO program, help downloading the program, and information about how to contact the project team in case of questions (Multimedia Appendix 1). Immediately following the telephone conversation, participants received an email from the project team member they had just spoken with, containing a form with EPIO program guidance and recommendations for use (Multimedia Appendix 2). No further contact with the project team took place unless the participants encountered technical issues and called the project telephone for help. All participants could contact the project team via a study-specific telephone for questions or technical assistance during regular office hours (ie, workdays 9 AM to 3 PM). EPIO program use was automatically logged, encrypted, and stored through the secure TSD research server. To explore participants’ perceived experiences with the use of the EPIO program, including the lack of follow-up during the study period, semistructured interviews were conducted postintervention with a subsample of EPIO_NoFollowUp_ participants after they had completed the 12-month outcome measures.

#### RCT: EPIO_BlendedCare_ and Care-As-Usual Control Group

Following informed consent and completion of baseline measures, participants in the RCT were randomized to either the intervention (ie, here called EPIO_BlendedCare_) or the control group [31]. The EPIO_BlendedCare_ group received a face-to-face introduction session with an introduction to and rationale for the EPIO program, help downloading EPIO from the Apple App Store or Google Play Store, and semistructured guidance on how to use the program. In addition, the blended care group received follow-up telephone calls from project team members at approximately 3 and 7 weeks after the introduction session to see how things were going (ie, standard questions about their program progress and whether they had any program-related questions) [31]. Poststudy interviews were also conducted with the EPIO_BlendedCare_ group, with findings pointing to positive changes in cognition, coping, and engagement [32].

The care-as-usual control group had no contact with the project team apart from, like all groups, receiving reminders about completion of outcome measures [31]. All participants were given continued access to the EPIO program poststudy if interested.

### The EPIO Intervention Program

**Figure 2:**
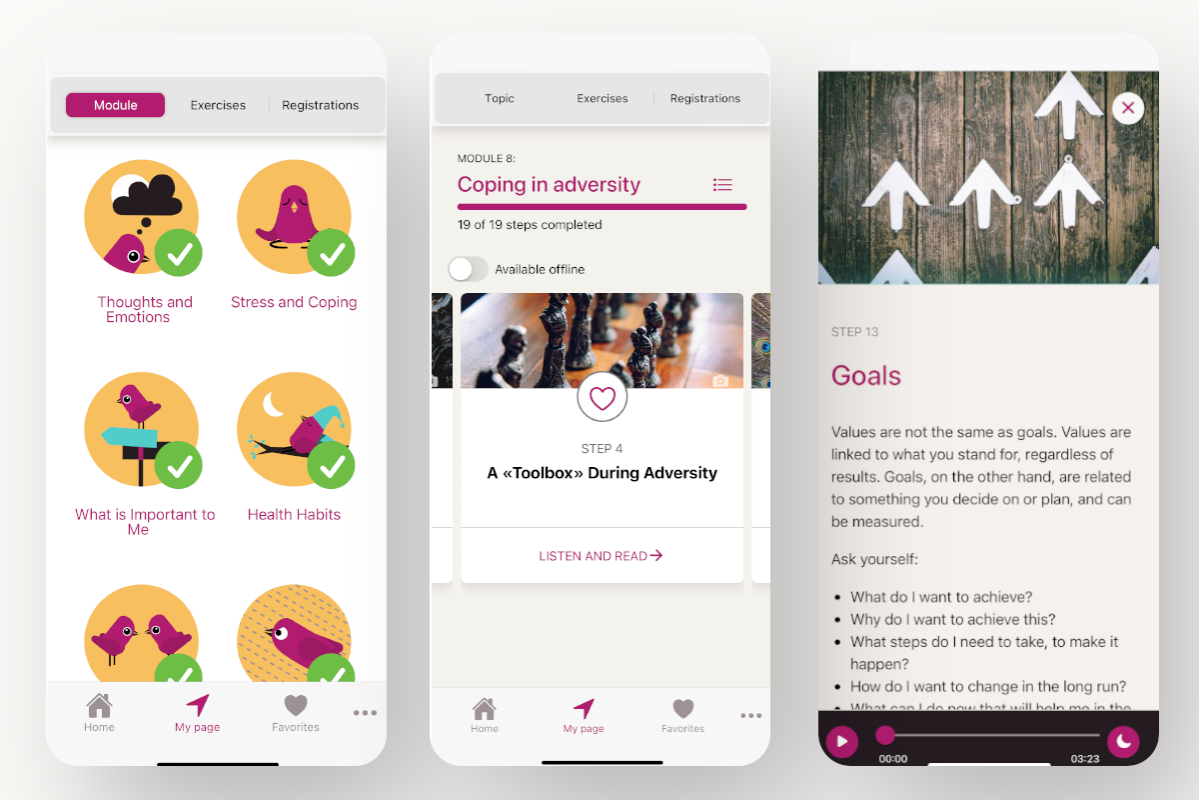
Examples of EPIO screenshots: modules, coping in adversity, and goals.

**Figure 3:**
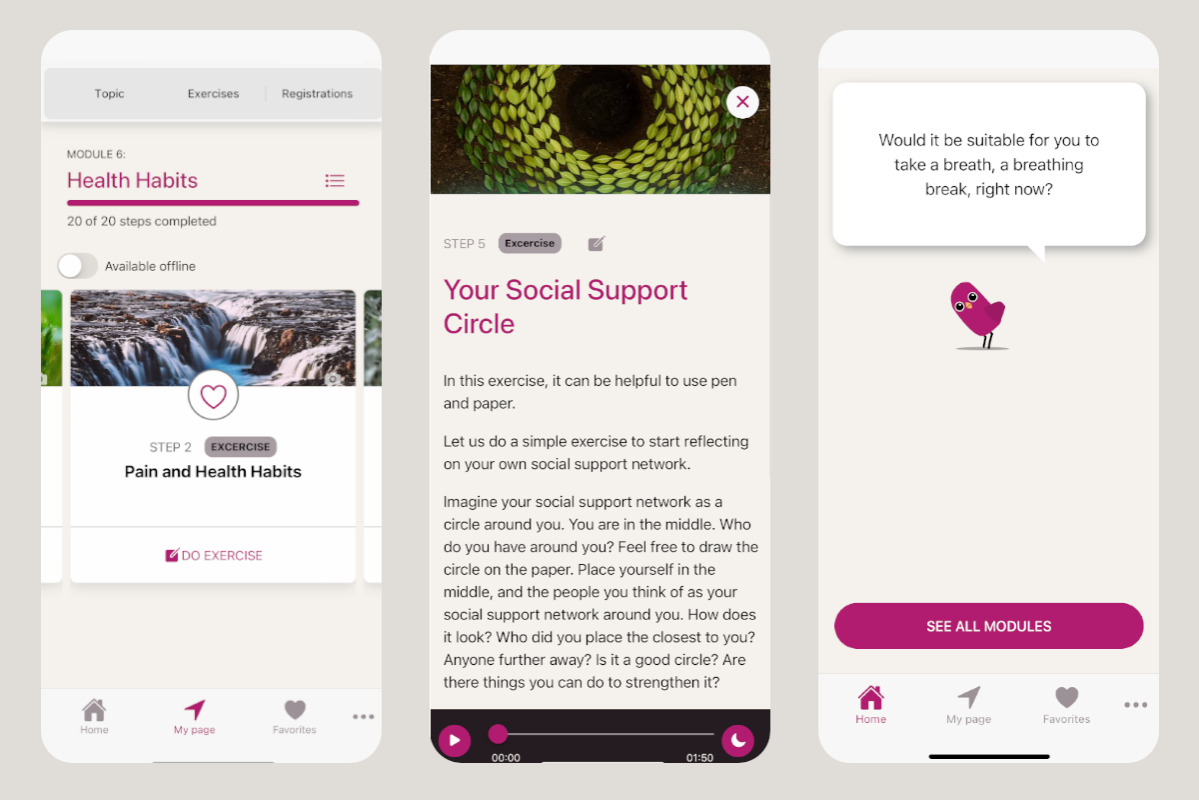
Examples of EPIO screenshots: health habits, social support, and EPIOS bird message.

The EPIO program consists of 9 CBT-based modules with aspects of ACT, centers on established pain self-management features, and each module contains a combination of psychoeducational content and exercises (eg, diaphragmatic breathing, visualization, mindfulness, progressive muscle relaxation, and thought challenging) [16,32]. The modules include the following main topics: (1) about pain, (2) activity pacing, (3) thoughts and emotions, (4) stress and coping, (5) what is important to me, (6) health habits, (7) communication and social support, (8) coping in adversity, and (9) reflections and the road ahead [16,32]. An animated bird (ie, the EPIOS avatar) accompanies users throughout the program. Further details related to functionalities and the design and development of EPIO can be found in existing publications [16,25,26]. Figures 2 and 3 provide screenshot examples from the EPIO program.

### Data Collection and Outcome Measures

#### Overview

Outcome measures were completed digitally through TSD at baseline and at 3, 6, and 12 months. All participants also completed a study-specific sociodemographic and disease-related questionnaire at baseline, including options to self-report pain conditions. As patients living with chronic pain often describe having more than 1 pain condition or painful area, more than 1 pain condition could be reported. A sequential mixed methods design was used, including quantitative data collection (ie, at baseline and at 3, 6, and 12 months) followed by qualitative interviews conducted after completion of the 12-month outcome measures.

#### Psychosocial Outcome Measures

##### Primary Outcome

Pain interference (ie, primary outcome) was measured with the short version of the Brief Pain Inventory (BPI) [40]. The BPI consists of 7 items assessing the impact of pain on daily function (ie, interference) and 4 items measuring pain severity. Scores range from 0 to 10, and higher scores indicate increased interference and pain severity. The BPI has acceptable construct validity (ie, a moderately strong relationship, *r*>0.5, between well-validated pain measures and BPI scale scores) and reliability coefficients for the BPI Severity and Interference scales ranging from 0.82 to 0.95 in samples of patients with arthritis and lower back pain [40]. The BPI has been validated in a Norwegian chronic pain population [41].

##### Secondary Outcomes

Symptoms of anxiety and depression were measured with the Hospital Anxiety and Depression Scale (HADS) [42]. The HADS consists of 14 items measuring symptoms of anxiety (7 items) and depression (7 items). Scores range from 0 to 21 for both subscales, with higher scores indicating higher symptoms of anxiety or depression. The scale has acceptable validity (ie, correlation between 0.60 and 0.80) and reliability (ie, Cronbach α ranging from 0.67 to 0.93) [43] and has been validated among people receiving primary care in Norway and in the Norwegian general population [44].

Self-regulatory fatigue was measured with the Self-Regulatory Fatigue Scale-18 (SRF-18) [45], an 18-item scale measuring self-regulatory capacity with cognitive, emotional, and behavioral components of self-regulation. Scores range from 18 to 90, and higher scores indicate higher self-regulatory fatigue. The SRF-18 has acceptable internal consistency and reliability (ie, Cronbach α ranging from 0.81 to 0.88) [45,46] and has been validated among people with cancer in Norway [46,47].

HRQoL was measured using the RAND 36-Item Health Survey (RAND-36), a noncommercial version of the 36-Item Short Form Health Survey [48,49]. The scale consists of 36 items measuring physical, role, emotional, cognitive, and social function, as well as physical, general, and global health. Scores range from 0 to 100, with higher scores indicating higher HRQoL. The scale has acceptable internal consistency and reliability [50] and has been validated in a Norwegian population with chronic pain, with acceptable to excellent reliability (ie, Cronbach α ranging from 0.74 to 0.91) [49].

Pain catastrophizing was measured with the Pain Catastrophizing Scale (PCS) [51]. The scale has 13 items measuring catastrophic thinking and maladaptive responses to pain and 3 subscales assessing helplessness, magnification, and rumination. Scores range from 0 to 52, with higher scores indicating a greater presence of catastrophic thoughts and feelings about pain. The scale has acceptable internal consistency and reliability (ie, Cronbach α=0.92) [52] and has been validated in a Norwegian population with chronic pain [53].

Pain acceptance was measured with the Chronic Pain Acceptance Questionnaire-8 (CPAQ-8) short form [54]. The scale contains 8 items assessing pain willingness (4 items) and activity engagement (4 items) [54]. Scores range from 0 to 24, with higher scores indicating greater acceptance of pain. The scale has acceptable validity and reliability (ie, Cronbach α ranging from 0.77 to 0.89) [55] and has been validated in a Norwegian population with chronic pain [56].

#### Program Use

Program progress and frequency of use were collected automatically and stored through the secure TSD research server. All data collection was in accordance with existing safety and privacy regulations.

### Sample and Statistical Power Estimates

Sample size in the RCT (ie, EPIO_BlendedCare_ and care-as-usual control) [31] was based on studies with comparable samples [57,58] reporting Cohen *d* effect sizes of 0.30 to 0.40 on the primary outcome, pain interference. Estimating an α of .05% and 80% power, a sample size of 200 participants was required to detect *d*=0.4. To account for attrition and provide adequate power for secondary analyses, the total RCT study sample included 266 participants [31]. Sample size in the EPIO_NoFollowUp_ group was calculated based on the anticipated change of 0.6 in the control group and a slightly greater change of 0.75 points in the EPIO_NoFollowUp_ group from baseline to the 12-month follow-up, with a common SD of 0.3. To show a between-group difference in change of 0.15 as statistically significant, a sample of 64 participants in each group was required. Accounting for at least 10% attrition, a sample size of 74 participants in the EPIO_NoFollowUp_ group was estimated.

### Statistical Analyses

Sociodemographic characteristics and user patterns were summarized with means and SDs for normally distributed variables and medians and IQRs for variables with skewed distributions. Categorical data were presented as counts and percentages. All analyses were conducted according to intention-to-treat principles, including all participants regardless of EPIO intervention use (ie, use in the EPIO_NoFollowUp_ and EPIO_BlendedCare_ groups). To explore potential between-group differences in outcome measures, the following analyses were performed:

- Step 1: baseline sociodemographic variables (eg, age, sex, and marital status) were compared across all 3 study groups to identify possible confounders. The distribution of continuous variables was compared using the nonparametric Kruskal-Wallis test, and categorical data were compared using chi-square tests. No between-group differences were revealed.
- Step 2: data were analyzed using generalized linear models (GLMs) for repeated measures with an identity link function and an unstructured covariance matrix to avoid imposing any parametric structure on the data. Time (ie, measurement points), group (EPIO_NoFollowUp_ vs control group and EPIO_NoFollowUp_ vs EPIO_BlendedCare_), and the interaction term (group × time) were entered as fixed effects, and study ID number (ie, a participant identifier) was entered as a random effect. As no statistically significant differences were observed between the 3 groups for sociodemographic variables (eg, age, sex, and marital status), as described in step 1, no variables were included in the intention-to-treat analysis as possible confounders. All measured time points were included in the analysis.
- Step 3: to explore how education level affected outcomes, GLMs, as described above, were fitted separately for EPIO_NoFollowUp_ and EPIO_BlendedCare_ with time × education level as an interaction term. Results from the GLMs are presented as estimated mean differences (MDs) between groups with 95% CIs and effect sizes (ESs) computed using Cohen *d*.

Analyses comparing program use between EPIO_NoFollowUp_ and EPIO_BlendedCare_ were performed using the independent-samples Mann-Whitney *U* test and chi-square test. There were no missing data at the item level in the completed outcome measures, as participants completed outcome measures digitally and could not proceed without completing all questions. Participants completing all outcome measures at the 3-, 6-, or 12-month assessment were referred to as “responders,” whereas those not completing outcome measures were referred to as “nonresponders.” To compare responders and nonresponders (ie, sensitivity analysis) on baseline sociodemographic characteristics (ie, age, sex, marital status, education, employment, disability benefits, income, and years living with pain), the chi-square test for categorical variables and the Mann-Whitney Wilcoxon test was used for continuous variables. *P* values <.05 were considered statistically significant. In addition, the Little MCAR (missing completely at random) test [59] was used to compare all outcome measures between responders and nonresponders. Findings from these comparisons were not statistically significant at any assessment point (*P<*.98); thus, the missingness was considered to be completely at random, and multiple imputations were therefore not needed. As all analyses were considered exploratory and hypothesis generating, findings are presented as unadjusted *P* values and 95% CIs without multiplicity correction, consistent with methodological and regulatory guidance for exploratory work and focused on point estimates and their precision (eg, 95% CIs rather than *P* values) [60,61]. Statistical analyses were conducted using SPSS (version 29; IBM Corp) and Stata (version 19; StataCorp LLC).

### Thematic Analyses

While quantitative data were collected to explore the potential effect of program access (ie, change from baseline in pain interference, pain severity, depression, anxiety, self-regulatory fatigue, HRQoL, pain catastrophizing, and pain acceptance) as well as actual program use (eg, program progress and frequency of use), qualitative data were collected primarily to explore participants’ perceived experiences with use of the EPIO program, including the lack of follow-up during the study period.

Prior to conducting the qualitative interviews in this study, a study-specific matrix was used to strive for a representative sample of the EPIO_NoFollowUp_ study population in terms of sex, age, years living with pain, and program progression (ie, low, moderate, and high) at 12 months. A subset of participants completing the 12-month follow-up outcome measurements in the EPIO_NoFollowUp_ group was subsequently identified and invited to participate in a poststudy semistructured interview to explore aspects of program use and impact. As no new information emerged after 15 interviews, data collection was concluded (ie, saturation). Qualitative data derived from the postintervention interviews were analyzed using a rapid analysis inspired by Gale et al [62]. Coauthor EFN read all transcripts to achieve a holistic impression, and data for each participant were categorized based on themes from the semistructured interview guide, including codes and quotes. All codes were then merged into a Microsoft Excel spreadsheet, sorted into subthemes, and the draft was reviewed and modified based on discussions between coauthors EFN and EB [62]. Illustrative quotes were subsequently compiled. Coauthors EFN, EB, and LSN further discussed the findings until reaching and securing consensus.

### Ethical Considerations

The study was approved by the Regional Committee for Medical and Health Research Ethics (REK; 2018/8911) and the Hospital Privacy Protection Committee (Personvernombudet; PVO) Institutional Review Board equivalent (2017/6697), and followed national and institutional privacy regulations and guidelines for data storage and handling. Prior to participant inclusion, the study was registered at ClinicalTrials.gov (NCT03705104). Study methods and results are reported in accordance with the CONSORT (Consolidated Standards of Reporting Trials) checklist (Multimedia Appendix 3) [63].

Signed informed consent forms and contact information were stored separately from study data in line with institutional regulations. Outcome measures were collected digitally through the secure TSD platform, and all personally identifiable information was deidentified before study data were exported to a local secure server for further analysis. Participants received no compensation for study participation.

## Results

### Sample Description

**Figure 4:**
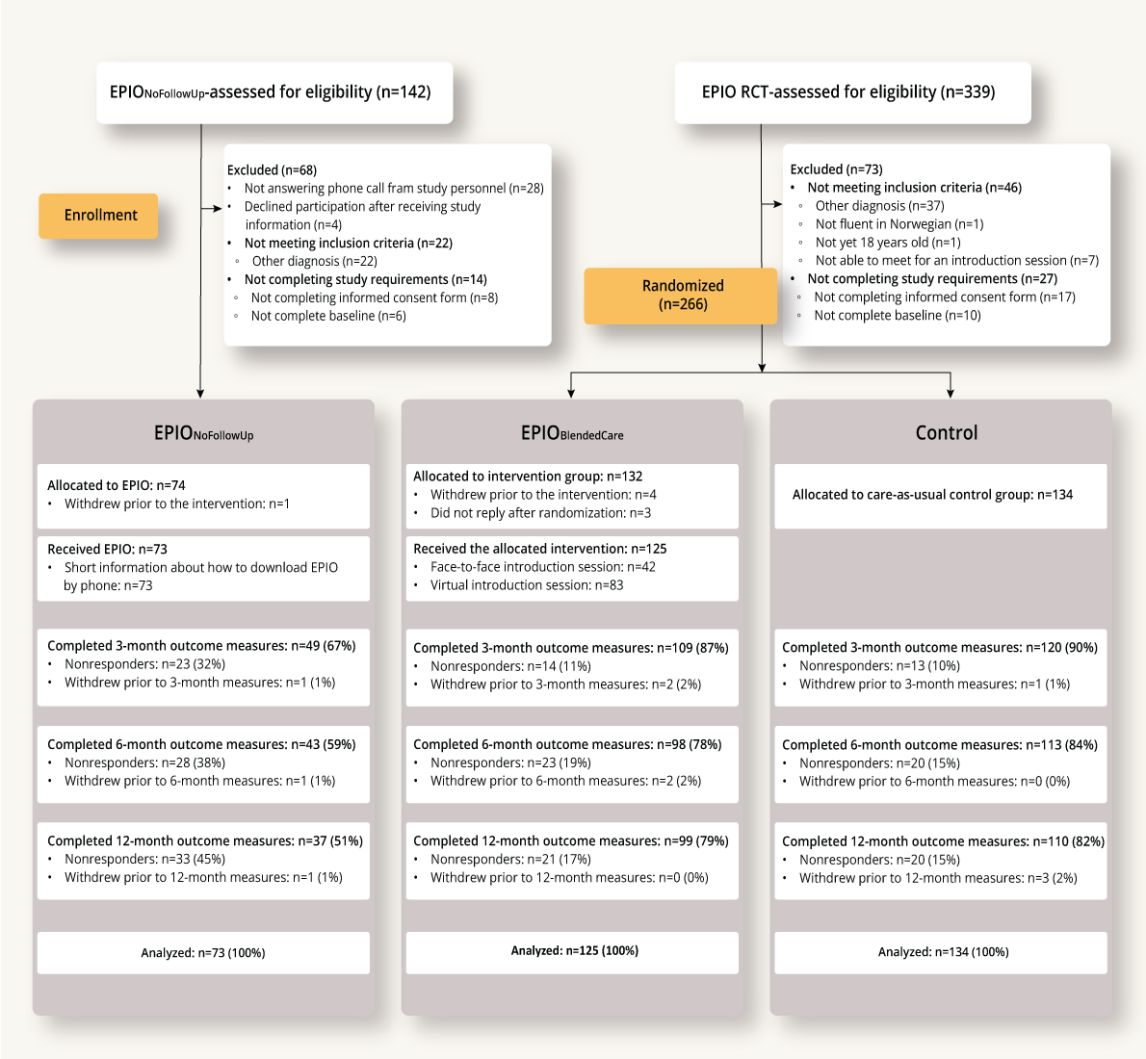
Trial recruitment and participant flowchart overview.

A total of 74 participants living with chronic pain were enrolled in the EPIO_NoFollowUp_ group, with 1 participant withdrawing prior to receiving the EPIO program, resulting in n=73 for the group. In the RCT, 266 participants were enrolled, with 7 participants withdrawing or failing to respond prior to the introduction session, resulting in n=259 (intervention group, n=125; control group, n=134) [30,31]. Figure 4 provides the trial recruitment and participant flowchart overview.

Of the participants in the EPIO_NoFollowUp_ group, 15 were interviewed after completion of the 12-month outcome measures. A total of 25 participants in the EPIO_BlendedCare_ group were previously interviewed after study completion [32]. The total number of participants in the EPIO_NoFollowUp_ and RCT (ie, EPIO_BlendedCare_ and care-as-usual control) groups was N=332, with a median age of 49 (IQR 39-55) years at inclusion, and participants were primarily female (271/332, 82%). Most participants reported being on sick leave or receiving disability benefits (232/332, 70%) at the time of enrollment and reported having lived with pain for 10 years or longer (201/332, 61%). No statistically significant differences in sociodemographic characteristics were observed at baseline, although participants differed somewhat in self-reported pain conditions (ie, participants in the EPIO_NoFollowUp_ group reported a higher percentage of nonspecific disc disorders and the category “other,” as well as a lower percentage of fibromyalgia, compared with the 2 RCT groups) and years living with pain. Sensitivity analysis showed no statistically significant differences between responders and nonresponders regarding sociodemographic characteristics (ie, age, sex, marital status, education, disability benefits, income, and years living with pain), except for sex at 3 months, when a significantly larger proportion of males (ie, considering that only 12 males were included in total) dropped out of the EPIO_NoFollowUp_ group, and for marital status at 12 months, when more single or divorced participants dropped out (Multimedia Appendix 4). Table 1 provides baseline sociodemographic and disease-related characteristics.

**Table 1:** Baseline sociodemographic and disease-related characteristics.

Characteristics			EPIO_NoFollowUp_ (n=73)		EPIO RCT^a^ (n=259)				*P* value	
					EPIO_BlendedCare_ (n=125)		Control (n=134)			
Age (years), median (IQR; range)			48 (38.5-56.5; 26-74)		50 (41-56; 26-74)		48 (38-53; 22-78)		.07	
**Sex, n (%)**										.77
	Female	61 (83.6)		103 (82.4)		107 (79.9)				
	Male	12 (16.4)		22 (17.6)		27 (20.1)				
**Marital status, n (%)**										.54
	Married or cohabitating	50 (68.5)		79 (63.2)		93 (69.4)				
	Single or divorced	23 (31.5)		46 (36.8)		41 (30.6)				
**Education, n (%)**										.81
	Elementary or high school	31 (42.4)		49 (39.2)		59 (44)				
	University or college ≤4 years	28 (38.4)		55 (44)		49 (36.6)				
	University or college >4 years	14 (19.2)		21 (16.8)		26 (19.4)				
**Employment, n (%)**										.50
	Full-time or part-time	22 (30.1)		30 (24)		27 (20.1)				
	Sick leave or disability benefits	46 (63)		86 (68.8)		100 (74.6)				
	Retired or others	5 (6.8)		9 (7.2)		7 (5.2)				
100 % disability benefits, n (%)			24 (32.9)		45 (36.6)		33 (24.6)			
**Income status (€)^b^, n (%)**										.84
	<40,000	12 (16.4)		28 (22.4)		26 (19.4)				
	40,000-60,000	13 (17.8)		19 (15.2)		22 (16.4)				
	60,000-80,000	16 (21.9)		32 (25.6)		31 (23.1)				
	80,000-100,000	13 (17.8)		24 (19.2)		31 (23.1)				
	>100,000	19 (26.0)		22 (17.6)		24 (17.9)				
**Self-reported pain conditions^c^, n (%)**										
	Unspecified musculoskeletal pain	11 (15)		27 (24.1)		32 (26)		.19		
	Unspecified disc disorder	27 (37)		15 (13.4)		16 (13)		<.001		
	Osteoarthritis	12 (16.4)		22 (19.6)		26 (21.1)		72		
	Rheumatoid arthritis	14 (19.2)		16 (14.3)		14 (11.5)		.32		
	Fibromyalgia	18 (24.7)		53 (47.3)		54 (43.9)		.006		
	Neuropathic pain	10 (13.7)		10 (8.9)		9 (7.3)		.33		
	Post injury or surgery	9 (12.3)		13 (11.6)		11 (8.9)		.71		
	Other	28 (38.4)		25 (22.5)		18 (14.6)		<.001		
**Years living with pain, n (%)**										.11
	<3	7 (9.6)		12 (9.6)		22 (16.4)		.18		
	3-5	8 (11)		7 (5.6)		17 (12.7)		.14		
	5-10	15 (20.5)		19 (15.2)		24 (17.9)		.62		
	>10	43 (58.9)		87 (69.6)		71 (53)		.02		

^a^RCT: randomized controlled trial.

^b^€1=US $1.16 as of summer 2026; €1=NOK 10.98 (Norwegian kroner).

^c^Participants could report having several types of self-reported conditions.

### Between-Group Differences

Results from the comparison of the EPIO_NoFollowUp_ group and the care-as-usual control group showed significant between-group differences in favor of the EPIO_NoFollowUp_ group in terms of improved HRQoL subscale bodily pain at 6 months (MD 5.7, 95% CI 0.4-11.1; *P*=.04; ES 0.15) and general health at 6 months (MD 5.7, 95% CI 0.8-10.5; *P*=.02; ES 0.16), reduced pain catastrophizing for the helplessness subscale at 6 months (MD –2.1, 95% CI –3.8 to –0.4; *P*=.02; ES 0.17), and improved chronic pain acceptance for the willingness subscale at 3 (MD 0.8, 95% CI 0.0-1.6; *P*=.046; ES 0.14) and 12 months (MD 1.4, 95% CI 0.2-2.7; *P*=.03; ES 0.15) and activity engagement at 3 months (MD 1.0, 95% CI 0.2-1.9; *P*=.02; ES 0.17), as well as total chronic pain acceptance at 3 months (MD 1.8, 95% CI 0.5-3.1; *P*=.007; ES 0.19). Certain trends toward between-group differences in favor of the EPIO_NoFollowUp_ group were also noted. Tables 2 and 3 provide outcome measure group comparisons at baseline and at 3, 6, and 12 months.

Results from the comparison of the EPIO_NoFollowUp_ group and the EPIO_BlendedCare_ group revealed only 1 significant between-group difference, with a statistically significant reduction in self-regulatory fatigue (MD –2.9, 95% CI 0.3-5.4; *P*=.03; ES 0.16) at 3 months in favor of the EPIO_BlendedCare_ group. Details are provided in Multimedia Appendix 5.

**Table 2:** Comparison I of outcome measures in the EPIO_NoFollowUp_ and EPIO_BlendedCare_ groups^a^ at baseline and at 3, 6, and 12 months.

Measures				EPIO_NoFollowUp_ (n=73)			EPIO RCT^b^ control group (n=134)			Between-group differences			
				Mean (95% CI)			Mean (95% CI)			MD^c^ (95% CI)			*P* value
**Pain interference (BPI^d^)^e^**													
	Baseline		5.0 (4.5 to 5.5)			5.4 (5.1 to 5.8)							
	3 months		5.1 (4.5 to 5.7)			5.1 (4.7 to 5.4)			0.4 (−0.1 to 1.9)			.10	
	6 months		4.4 (3.8 to 5.0)			4.9 (4.5 to 5.3)			0.0 (−0.6 to 0.6)			.97	
	12 months		4.4 (3.8 to 5.1)			4.8 (4.4 to 5.2)			0.0 (−0.7 to 0.8)			.91	
**Pain severity (BPI^d^)^f^**													
	Baseline		5.0 (4.7 to 5.4)			5.3 (5.0 to 5.5)							
	3 months		5.0 (4.6 to 5.4)			5.2 (5.0 to 5.5)			−0.0 (−0.4 to 0.4)			.91	
	6 months		4.6 (4.1 to 5.1)			5.2 (4.9 to 5.5)			−0.4 (−0.9 to 0.1)			.15	
	12 months		4.9 (4.4 to 5.3)			5.1 (4.8 to 5.5)			−0.0 (−0.5 to 0.4)			.86	
**Anxiety (HADS-A^g^)**													
	Baseline		8.1 (7.2 to 9.0)			8.0 (7.2 to 8.7)							
	3 months		7.4(6.5 to 8.3)			7.4 (6.6 to 8.1)			−0.1 (−1.1 to 0.9)			.89	
	6 months		7.6 (6.5 to 8.8)			7.5 (6.7 to 8.3)			0.0 (−1.3 to 1.2)			.95	
	12 months		7.8 (6.9 to 8.8)			7.8 (6.9 to 8.6)			0.0 (−1.2 to 1.1)			.95	
**Depression (HADS-D^h^)**													
	Baseline		7.3 (6.5 to 8.1)			7.1 (6.5 to 7.7)							
	3 months		6.7 (5.7 to 7.6)			6.8 (6.2 to 7.5)			−0.3 (−1.3 to 0.6)			.51	
	6 months		5.8 (4.8 to 6.8)			6.6 (6.0 to 7.3)			−1.0 (−2.0 to 0.0)			.60	
	12 months		6.7 (5.7 to 7.7)			6.9 (6.1 to 7.6)			−0.4 (−1.4 to 0.7)			.51	
**Self-regulatory fatigue (SRF-18^i^)**													
	Baseline		55.6 (52.6 to 58.2)			55.7 (53.8 to 47.7)							
	3 months		55.4 (52.6 to 58.2)			55.3 (53.4 to 57.2)			0.2 (−2.4 to 2.8)			.90	
	6 months		54.1 (51.2 to 57.0)			55.1 (53.2 to 57.0)			−0.9 (−3.7 to 1.8)			.50	
	12 months		54.7 (51.5 to 57.9)			55.1 (53.0 to 57.2)			−0.3 (−3.7 to 3.1)			.86	
**HRQoL^j^ (RAND-36^k^)**													
	**Physical functioning**												
		Baseline	57.9 (52.9 to 63.0)		54.7 (50.9 to 58.4)								
		3 months	60.5 (54.8 to 66.2)		57.3 (53.3 to 61.2)			0.0 (−4.3 to 4.3)			.99		
		6 months	64.1 (58.4 to 69.6)		60.1 (56.0 to 64.1)			−0.7 (−4.2 to 5.5)			.79		
		12 months	63.3 (56.7 to 69.8)		60.8 (56.9 to 64.7)			−0.8 (−5.9 to 4.3)			.76		
	**Role-physical**												
		Baseline	18.5 (11.2 to 25.8)		11.0 (7.6 to 14.4)								
		3 months	26.9 (18.0 to 35.8)		19.4 (14.0 to 24.9)			0.0 (−10.5 to 10.5)			.99		
		6 months	31.3 (20.8 to 41.8)		18.5 (13.0 to 24.0)			5.3 (−5.5 to 16.1)			.33		
		12 months	27.8 (17.7 to 37.9)		18.6 (13.2 to 24.1)			1.7 (−9.2 to 12.6)			.76		
	**Bodily pain**												
		Baseline	26.2 (22.3 to 30.2)		26.9 (24.4 to 29.4)								
		3 months	31.9 (26.5 to 37.4)		28.9 (25.6 to 32.2)			3.7 (−2.4 to 9.7)			.24		
		6 months	35.7 (30.4 to 41.0)		30.6 (27.5 to 33.7)			5.7 (0.4 to 11.1)			.04		
		12 months	33.9 (28.3 to 39.6)		30.2 (26.3 to 34.0)			4.5 (−1.9 to 10.8)			.17		
	**General health**												
		Baseline	37.7 (33.1 to 42.3)		36.5 (33.1 to 39.8)								
		3 months	38.5 (33.2 to 43.7)		38.2 (34.9 to 41.6)			−1.0 (−6.0 to 4.1)			.71		
		6 months	44.7 (40.0 to 49.4)		37.8 (34.0 to 41.6)			5.7 (0.8 to 10.5)			.02		
		12 months	41.2 (35.8 to 46.5)		36.0 (33.2 to 40.7)			3.1 (−2.3 to 8.5)			.27		
	**Vitality**												
		Baseline	24.4 (20.1 to 28.7)		24.7 (21.5 to 28.0)								
		3 months	30.4 (24.6 to 36.2)		26.9 (23.3 to 30.4)			3.9 (−2.0 to 9.8)			.19		
		6 months	30.9 (25.7 to 36.1)		27.8 (24.2 to 31.4)			3.5 (−2.3 to 9.2)			.24		
		12 months	25.1 (19.5 to 30.8)		25.8 (22.4 to 29.3)			−0.3 (−6.3 to 5.7)			.91		
	**Social functioning**												
		Baseline	45.4 (39.7 to 51.1)		45.7 (41.7 to 49.7)								
		3 months	48.8 (42.4 to 55.1)		50.6 (45.8 to 55.3)			−1.5 (−8.7 to 5.8)			.69		
		6 months	53.2 (46.3 to 60.1)		51.2 (46.8 to 55.6)			2.3 (−5.4 to 10.1)			.55		
		12 months	52.9 (45.3 to 60.6)		49.2 (44.9 to 53.5)			4.0 (−4.2 to 12.2)			.34		
	**Role-emotional**												
		Baseline	53.4 (43.5 to 63.4)		55.7 (45.1 to 60.4)								
		3 months	56.0 (45.1 to 66.8)		54.7 (46.5 to 62.8)			0.6 (−15.1 to 16.4)			.94		
		6 months	51.2 (39.4 to 63.1)		49.7 (41.9 to 57.5)			0.9 (−13.7 to 15.4)			.91		
		12 months	51.2 (37.1 to 65.2)		51.4 (43.5 to 59.3)			−1.0 (−19.2 to 17.3)			.92		
	**Mental health**												
		Baseline	62.5 (58.6 to 66.3)		64.0 (61.0 to 67.3)								
		3 months	64.0 (59.8 to 68.3)		62.9 (59.5 to 66.4)			2.6 (−2.0 to 7.2)			.27		
		6 months	65.1 (60.1 to 70.1)		63.8 (60.2 to 67.3)			2.8 (−3.7 to 9.4)			.39		
		12 months	63.7 (58.6 to 68.8)		62.0 (58.4 to 65.7)			3.2 (−2.6 to 9.0)			.28		

^a^Estimated marginal means from generalized linear mixed models.

^b^RCT: randomized controlled trial.

^c^MD: mean difference.

^d^BPI: Brief Pain Inventory.

^e^Subscale of the Brief Pain Inventory (score range 0-10; a higher score indicates higher interference in life).

^f^Subscale of the Brief Pain Inventory (score range 0-10; a higher score indicates higher severity).

^g^HADS-A: Hospital Anxiety and Depression Scale-Anxiety subscale (score range 0-21; a higher score indicates a higher degree of anxiety).

^h^HADS-D: Hospital Anxiety and Depression Scale-Depression subscale (score range 0-21; a higher score indicates a higher degree of depression).

^i^SRF-18: Self-Regulatory Fatigue Scale-18 (score range 18-90; a higher score indicates higher self-regulatory fatigue).

^j^HRQoL: health-related quality of life.

^k^RAND-36: RAND 36-Item scale (score range 0-100; a higher score indicates higher emotional well-being).

**Table 3:** Comparison II of outcome measures in the EPIO_NoFollowUp_ and EPIO_BlendedCare_ groups^a^ at baseline and at 3, 6, and 12 months.

Measures				EPIO_NoFollowUp (_n=73)		EPIO RCT^b^ control group (n=134)		Between-group differences		
				Mean (95% CI)		Mean (95% CI)		MD^c^ (95% CI)		*P* value
**Pain catastrophizing (PCS^d^)**										
	**Rumination**									
		Baseline	7.6 (6.6 to 8.6)		8.1 (7.5 to 8.8)					
		3 months	6.6 (5.7 to 7.6)		7.8 (7.2 to 8.5)		−0.7 (−1.8 to 0.4)		.20	
		6 months	6.6 (5.4 to 7.8)		7.7 (7.0 to 8.4)		−0.6 (−1.8 to 0.6)		.34	
		12 months	6.2 (5.0 to 7.4)		7.3 (6.5 to 8.1)		−0.6 (−2.0 to 0.8)		.42	
	**Magnification**									
		Baseline	3.8 (3.2 to 4.3)		3.7 (3.2 to 4.2)					
		3 months	3.2 (2.6 to 3.8)		3.7 (3.2 to 4.1)		−0.5 (−1.2 to 0.3)		.22	
		6 months	3.4 (2.8 to 4.1)		3.6 (3.1 to 4.0)		−0.2 (−0.9 to 0.6)		.64	
		12 months	3.0 (2.3 to 3.6)		3.6 (3.1 to 4.0)		−0.6 (−1.4 to 0.2)		.14	
	**Helplessness**									
		Baseline	9.9 (8.6 to 11.1)		9.5 (8.6 to 10.4)					
		3 months	8.5 (7.1 to 9.8)		9.3 (8.4 to 10.1)		−1.2 (−2.7 to 0.4)		.13	
		6 months	7.4 (5.9 to 8.9)		9.1 (8.3 to 10.0)		−2.1 (−3.8 to −0.4)		.02	
		12 months	8.3 (6.8 to 9.8)		9.0 (8.0 to 10.0)		−1.0 (−2.8 to 0.8)		.27	
	**PCS total**									
		Baseline	21.3 (18.7 to 23.8)		21.4 (19.5 to 23.1)					
		3 months	18.3 (15.8 to 20.9)		20.8 (19.1 to 22.5)		−2.4 (−5.0 to 0.3)		.08	
		6 months	17.4 (14.5 to 20.3)		20.4 (18.6 to 22.3)		−2.9 (−6.1 to 0.2)		.07	
		12 months	17.5 (14.5 to 20.5)		19.8 (17.7 to −21.9)		−2.3 (−5.8 to 1.3)		.21	
**Chronic pain acceptance (CPAQ^e^)**										
	**Willingness**									
		Baseline	13.4 (12.8 to 14.0)		13.7 (13.2 to 14.2)					
		3 months	14.1 (13.6 to 14.7)		13.6 (13.2 to 14.1)		0.8 (0.0 to 1.6)		<.05	
		6 months	14.2 (13.5 to 14.9)		13.7 (13.2 to 14.1)		0.8 (−0.1 to 1.8)		.09	
		12 months	14.6 (13.6 to 15.7)		13.5 (13.0 to 14.0)		1.4 (0.2 to 2.7)		.03	
	**Activity engagement**									
		Baseline	13.7 (13.0 to 14.5)		13.7 (13.1 to 14.3)					
		3 months	14.6 (13.8 to 15.4)		13.6 (13.0 to 14.2)		1.0 (0.2 to 1.9)		<.02	
		6 months	14.9 (14.0 to 15.7)		14.0 (13.4 to 14.6)		0.8 (−0.2 to 1.9)		.13	
		12 months	14.0 (13.1 to 14.9)		14.2 (13.6 to 14.8)		−0.2 (−1.3 to 0.8)		.65	
	**CPAQ total**									
		Baseline	27.1 (26.0 to 28.3)		27.4 (26.5 to 28.3)					
		3 months	28.8 (27.7 to 29.9)		27.2 (26.3 to 28.1)		1.8 (0.5 to 3.1)		.01	
		6 months	29.0 (27.7 to 30.4)		27.7 (26.7 to 28.6)		1.6 (−0.1 to 3.4)		.07	
		12 months	28.6 (27.1 to 30.1)		27.7 (26.8 to 28.6)		1.1 (−0.7 to 3.0)		.22	

^a^Estimated marginal means from generalized linear mixed models.

^b^RCT: randomized controlled trial.

^c^MD: mean difference.

^d^PCS: Pain Catastrophizing Scale (score range 0-52; a higher score indicates higher catastrophizing).

^e^CPAQ: Chronic Pain Acceptance Questionnaire (score range 0-52; a higher score indicates a higher acceptance of pain).

### Program Use

Participants in the EPIO_NoFollowUp_ group used the EPIO program significantly less than those in the EPIO_BlendedCare_ group. Table 4 provides details. In the EPIO_NoFollowUp_ group, the median number of days of program use during the 12-month trial was 10 (IQR 3-24) days, compared with 30 (IQR 13-44) days in the EPIO_BlendedCare_ group. The same pattern was seen in module completion status, with 23% (17/73) completing ≥7 modules in the EPIO_NoFollowUp_ group at 12 months, vs 62% (78/125) in the EPIO_BlendedCare_ group (*P*<.001). Participants completing all modules accounted for 12% (9/73) in the EPIO_NoFollowUp_ group vs 45% (56/125) in the EPIO_BlendedCare_ group (*P*<.001).

**Table 4:** Program use in the EPIO_NoFollowUp_ vs EPIO_BlendedCare_ groups at 3, 6, and 12 months.

Use of EPIO				EPIO_NoFollowUp_ (n=73)		EPIO_BlendedCare_ (n=125)		*P* value
**At 3 months**								
	Individual days of EPIO use, median (IQR; range)		8 (2-17; 1-74)		21 (10-33.5; 1-85)		<.001^a^	
	Program module completion, median (IQR; range)		2 (0-3.5; 0-9)		6 (2-8; 0-9)		<.001^a^	
	Completed all program modules, n (%)		4 (6)		25 (20)		.005^b^	
	**Module completion, n (%)**							<.001^b^
		Low (0-2 modules completed)	47 (64)		35 (28)			
		Moderate (3-6 modules completed)	16 (22)		30 (24)			
		High (7-9 modules completed)	10 (14)		60 (48)			
**At 6 months**								
	Individual days of EPIO use, median (IQR; range)		9 (2-19.5; 1-138)		27 (11-37.5; 1-159)		<.001^a^	
	Program module completion, median (IQR; range)		2 (0-4; 0-9)		8 (3-9; 0-9)		<.001^a^	
	Completed all program modules, n (%)		9 (17)		44 (83)		<.001^b^	
	**Module completion, n (%)**							<.001^b^
		Low (0-2 modules completed)	43 (59)		28 (14)			
		Moderate (3-6 modules completed)	16 (22)		24 (19)			
		High (7-9 modules completed)	14 (19)		73 (58)			
**At 12 months**								
	Individual days of EPIO use, median (IQR; range)		10 (3-24; 1-210)		30 (13-44; 1-315)		<.001^a^	
	Program module completion, median (IQR; range)		2 (0-6; 0-9)		8 (3-9; 0-9)		<.001^a^	
	Completed all program modules, n (%)		9 (12)		56 (45)		<.001^b^	
	**Module completion, n (%)**							<.001^b^
		Low (0-2 modules completed)	43 (59)		27 (22)			
		Moderate (3-6 modules completed)	13 (18)		20 (16)			
		High (7-9 modules completed)	17 (23)		78 (62)			

^a^Independent-samples Kruskal-Wallis test.

^b^Chi-square test.

### Exploratory Analyses: Education Level

#### Overview

Analyses exploring the potential impact of education level on outcome measures showed a statistically significant impact of education level in the EPIO_NoFollowUp_ group. Adjusting for baseline values and comparing findings with those for participants with elementary or high school-level education showed statistically significant improvement for those with >4-year college or university education in terms of between-group differences for pain severity at 3 (B=–1.1, 95% CI –2.1 to 0.0; *P*=.04; ES 0.24) and 6 months (B=–1.5, 95% CI –2.6 to 0.5; *P*=.005; ES 0.33), for anxiety at 6 months (B=–3.1, 95% CI –5.6 to –0.6; *P*=.02; ES 0.28), for HRQoL subscale role-physical at 3 months (B=26.2, 95% CI 3.6-48.7; *P*=.02; ES 0.27), subscale vitality at 3 (B=15.7, 95% CI 3.1-28.4; *P*=.02; ES 0.29) and 6 (B=14.2, 95% CI 1.2-27.1; *P*=.03; ES 0.25) months, subscale mental health at 12 months (B=13.8, 95% CI 0.3-27.3; *P*=.045; ES 0.23), and pain catastrophizing subscale rumination at 6 (B=–3.0, 95% CI –5.7 to –0.4; *P*=.03; ES 0.26) and 12 (B=–3.4, 95% CI –6.3 to –0.6; *P*=.02; ES 0.27) months, as well as pain catastrophizing total at 6 (B=–7.2, 95% CI –13.8 to –0.5; *P*=.03; ES 0.25) and 12 (B=–7.6, 95% CI –14.7 to -0.5; *P*=.04; ES 0.25) months. Details are provided in Multimedia Appendix 6.

Few such indications of an impact of education level were detected for the EPIO_BlendedCare_ RCT intervention group, although statistically significant improvements in HRQoL subscale vitality were found in the group with ≤4 years of college education at 3 months (B=8.1, 95% CI 1.6-14.6; *P*=.02; ES 0.22) and for pain catastrophizing subscale helplessness at 3 months (B=2.0, 95% CI 0.1-4.0; *P*=.04; ES 0.18) among those with >4 years of college or university education, compared with those with elementary or high school-level education. Details are provided in Multimedia Appendix 6.

#### Program Use Based on Education Level

Education level appeared to also impact program use in terms of module completion. Estimated means (ie, change from the elementary or high school-level education group), for example, averaged 1.5 more modules completed at 3 months for those with ≥4 years of university or college-level education in the EPIO_NoFollowUp_ group (*P*<.001), compared with 0.1 more module in the EPIO_BlendedCare_ group. The same pattern was seen at 6 months (*P*=.002). Individual days of use were, however, significantly higher for participants with higher education in both intervention groups. Details are provided in Multimedia Appendix 7.

### Postintervention Interviews: EPIO_NoFollowUp_—Qualitative Analyses

#### Overview

Of participants meeting the interview inclusion criteria (ie, having completed 12-month outcome measures; n=37), 15 agreed to be interviewed, 16 declined, and the remaining 6 were not asked because of study matrix aspects. There were no statistically significant differences in sociodemographic and disease-related characteristics at baseline between the interviewed (n=15) and noninterviewed (n=58) participants. In addition, there were no significant differences in program use (ie, module completion progress and individual days used) between interviewed (n=15) and noninterviewed (n=22) participants completing the 12-month outcome measures. Participants completing the 12-month outcome measures (n=37) did, however, have significantly higher program use than those not completing the 12-month outcome measures (ie, not meeting interview inclusion criteria; n=36). Data analysis indicated data saturation, and the number of participants interviewed was considered sufficient for rich participant input [64].

The qualitative rapid analyses exploring perceived experiences with the use of EPIO, including the lack of follow-up during the 12 months, revealed that participants considered the content of EPIO informative, varied, and adapted to the perspectives of people living with pain, normalizing difficult topics. The program content was described as important public education, something all patients with pain should have as a tool, and something general practitioners could prescribe. Some even described the EPIO program as “medicine” in their lives. While about one-third of the interviewed participants stated that they had not missed having follow-up during the study, about two-thirds said they would likely have appreciated more follow-up. Three main themes related to EPIO program access were also identified, including reported changes in awareness, acceptance, and coping based on program access and use.

#### Regarding Follow-Up

The majority of participants interviewed (ie, about two-thirds) said that more follow-up from the project team would have been appreciated, with some stating that follow-up likely would have reminded them to use the EPIO program more, potentially making their use more motivating and leading to increased program use. Follow-up and being able to discuss EPIO were also described as possibly fostering greater ownership of the program. The need for social contact was also emphasized, with life with chronic pain being described as lonely and isolating. Some participants mentioned the importance of having a good relationship with and follow-up from their general practitioner or other health professionals, stating that it could have been nice to be able to discuss and learn alongside others. As one participant said:

I think [the app] provided more information than I have ever received from health care providers. And more information than I received during my pain rehabilitation stay, even though I probably got a lot of the same information there as well. I just feel that the [EPIO] app content was more wholesome and put into context. That’s why I think that if the app could be used together with someone, healthcare providers, the effect would be so much greater, for so many. Yes.

ID340

Participants claiming they had not missed having follow-up explained this as likely due to the EPIO program being user-friendly, easy to use, and easy to understand. One of them described preferring not to have to talk to many people, whereas others described appreciating being able to remain anonymous and not having to be the one “always complaining.” Many participants reported using reminders to remember to do relevant exercises during the day, and some described the program, including the animated EPIOS bird, as being a good friend and guide. Participants reported liking the flexible and independent approach provided by EPIO, and more than half of the interviewed participants reported doing breathing and relaxation exercises without opening the EPIO program itself after a while. Textbox 1 provides an overview of follow-up–related feedback from poststudy interviews with the EPIO_NoFollowUp_ group.

**Overview of follow-up–related feedback from the EPIO_NoFollowUp_ group.**

**Not missing follow-up, due to:**

- User-friendly program, easy to use and understand
- Helpful program reminders
- The program has become a friend
- Flexible and independent program use
- Not having to bother anyone else
- Using the program exercises without accessing the app

**Would have appreciated follow-up, which could have:**

- Enhanced motivation for use
- Served as reminders of program use
- Led to more use
- Enabled useful program discussions
- Met a need for social contact
- Enabled relationship with health professionals

#### Awareness

Participants reported having experienced increased awareness after using EPIO, particularly regarding their own thoughts and feelings, stating that they had also begun thinking differently about their pain after having access to the program. For example, some talked about learning to live with the pain, making calculated choices about their activities, and not letting the pain “take over.” Others described EPIO as having made them more mindful of their breathing, helped them become more intentional about using relaxation methods, and focused more on things they could control rather than things they could not impact. Multimedia Appendix 8 provides examples of EPIO_NoFollowUp_ participant quotes.

#### Acceptance

Some participants talked about noticing changes related to accepting their situation, describing having learned to accept the limitations induced by the pain and having become better at dealing with their pain. They reported experiencing a change of mind, having worked through their pain-related sense of loss and grief (eg, loss of tasks and roles), and then reaching acceptance and trying to focus on what is possible in life and how to live better with pain, rather than the many losses experienced due to pain. Multimedia Appendix 8 provides examples of quotes.

#### Coping

Participants described having gained helpful coping strategies through EPIO and highlighted breathing and relaxation exercises as particularly beneficial. They described using breathing and relaxation exercises to help calm down and for pain relief, taking a break, redirecting thoughts, and even falling asleep or going back to sleep when waking at night. Participants also reported having become better at setting boundaries and described learning about the importance of activity pacing and taking better care of themselves as helpful. Multimedia Appendix 8 provides examples of quotes.

## Discussion

### Overview

Findings show how the type of delivery model may impact the use of and benefit from digital self-management interventions. In the current study, a statistically significant impact was detected for the EPIO_NoFollowUp_ delivery model, compared with controls, in terms of improved HRQoL, reduced pain catastrophizing, and improved pain acceptance. Participants receiving the EPIO pain self-management program with no follow-up did, however, use the program significantly less than those receiving the EPIO_BlendedCare_ model, and the majority of participants receiving EPIO_NoFollowUp_ indicated that some form of follow-up would have been useful and appreciated.

The study also indicates how “one size does not fit all,” as people with a higher education level, despite having no follow-up, appeared to use the intervention more and benefit more (ie, in terms of improvement in pain severity, anxiety, HRQoL, and pain catastrophizing), compared with those with lower education levels. For participants receiving EPIO through the simple blended care model, formal education level did not seem to make a difference.

In line with the original RCT (ie, EPIO_BlendedCare_ vs care-as-usual control groups) [31], no significant between-group differences were detected for the primary outcome measure of pain interference with function. The findings did, however, also in line with results from the RCT [31], suggest more significant changes in psychological rather than physiological variables for the EPIO_NoFollowUp_ group compared with controls, likely at least partially due to the psychosocial self-management concept of the EPIO intervention [16,31].

### Access to a Digital Health Intervention Without Follow-Up

Even without follow-up (ie, EPIO_NoFollowUp_), people living with chronic pain having access to EPIO reported significantly improved HRQoL in terms of less bodily pain (ie, at 3 months) and improved general health (ie, at 6 months), as well as significantly reduced catastrophizing about pain in terms of feelings of helplessness (ie, at 6 months), compared with participants without access to EPIO (ie, care-as-usual controls). The findings were supported by qualitative input describing improved awareness related to their own thoughts, feelings, and how to live with the pain (ie, rather than fight it), as well as having acquired coping strategies that helped them deal with pain and the many pain-related aspects in new and beneficial ways. Findings also showed improved acceptance of pain (ie, improved activity engagement and pain acceptance total at 3 months, and improved willingness for pain acceptance at 12 months) compared with controls, and qualitative analyses supported this notion.

Access to digital pain self-management interventions, even without follow-up, may hence contribute to improved QoL and coping with pain in day-to-day life. Considering the potential decrease in impact from 6 to 12 months (ie, apart from willingness to accept pain), it is possible that actual improvement in activity engagement must take place for willingness to accept pain to occur. These findings indicate that access to a digital pain self-management intervention such as EPIO, even without follow-up, could contribute to pain acceptance and hence better QoL. For example, findings from an RCT administering a brief ACT intervention to public health workers experiencing chronic stress and pain indicated intervention benefits, including having fewer sick days and using less medical treatment, compared with controls [65].

Directly comparing participants in EPIO_NoFollowUp_ with those receiving EPIO_BlendedCare_ yielded only 1 significant between-group finding (ie, reduction in self-regulatory fatigue in favor of the EPIO_BlendedCare_ group at 3 months). With neither delivery model appearing superior to the other, this study supports research indicating that both guided and unguided digital interventions can be effective [66]. Results from the EPIO RCT did, however, reveal significant benefits for people with chronic pain having access to EPIO through a simple blended care model (ie, EPIO_BlendedCare_), compared with controls, including reduced anxiety, depression, self-regulatory fatigue, and pain catastrophizing, as well as improved HRQoL for the intervention group [31]. This points to the blended care delivery model, when compared with controls, as superior. The current findings are hence in line with those of a 3-arm RCT examining the effectiveness of guided and unguided digital ACT-based intervention for chronic pain [33], in which the guided version, compared with controls, showed a stronger impact than the unguided version, but with no major differences in terms of effect between the 2 intervention groups (ie, guided vs unguided) [33].

Postintervention qualitative findings (ie, using a sequential mixed methods approach) contributed to an enrichment of the study findings, identifying perceived experiences with the use of EPIO, despite the no-follow-up delivery model, to include changes in awareness, acceptance, and coping. These findings are in accordance with findings from the EPIO_BlendedCare_ group, where the identified main themes and ensuing subthemes included perceived changes in cognition (ie, subthemes insights and self-awareness, acceptance, and shifting focus), changes in coping (ie, subthemes pain, emotions, and activity pacing), and content functionality-specific engagement (ie, subthemes breathing and other mind-body exercises, thought-reflection exercises, and functionalities) [32].

### Program Use

Actual use and intervention adherence are crucial to induce effect, and when intervention engagement and adherence are low, the intended intervention effects are unlikely. The well-known ≤50% adherence challenges associated with digital health interventions are concerning [67,68], and studies point to the necessity of some form of guidance to enhance intervention adherence and hence impact [33-35].

This notion is supported by the current findings, with participants in the EPIO_NoFollowUp_ group clearly using the program significantly less (median 10, IQR 3-24 days of use) over the 12-month study period compared with those in the EPIO_BlendedCare_ group (median 30, IQR 13-44 days of use). Similarly, only 23% (17/73) of participants in the EPIO_NoFollowUp_ group completed ≥7 modules, compared with 62% (78/125) in the EPIO_BlendedCare_ group. While postintervention interviews identified EPIO as useful and easy to use, with highly relevant content, which is in line with previous qualitative findings [28,29,32,69] regardless of delivery method, a majority of participants in the EPIO_NoFollowUp_ group said they would have appreciated some form of contact and follow-up from health care providers or project team members.

Supporting recent research, these findings underline the importance of some form of guidance to foster intervention adherence. For example, a systematic review points to the necessity of reporting on engagement data to strengthen the development and effect of digital pain self-management interventions [70]. Other findings also support this notion. For example, in a meta-analysis examining the impact of guidance in mental health interventions, guidance apparently increased intervention completion, and completion rates were 12% higher in guided vs nonguided interventions [71].

Despite the apparent vital relationship between lack of follow-up and program use, several participants did describe having used the program exercises without using the EPIO app after initial practice, which highlights a form of use not captured through quantitative program use measures. This might also potentially explain the detection of some effects despite low intervention use in the EPIO_NoFollowUp_ group. Previous research has also shown that simply having access to a program or having a contact option can be helpful and contribute to health benefits [72,73].

### Potential Role of Education Level

Education level may impact benefit from health interventions. Higher education has, for example, been associated with profiting more from CBT-type approaches to self-management [74]. Participants with higher education in the EPIO_NoFollowUp_ group in the current study reported improvement in terms of pain severity, anxiety, HRQoL (ie, subscales role-physical, vitality, and mental health), and pain catastrophizing during the 12-month study, compared with those with an elementary or high school education. Formal education level has also been associated with intervention adherence and attrition [75], and participants with higher education in the EPIO_NoFollowUp_ group completed significantly more program modules compared with those with a lower education level.

Education level did not appear to influence outcomes or adherence to the same extent for participants receiving the EPIO_BlendedCare_ delivery model, although individual days of use were significantly higher among participants with higher education in both intervention groups. These findings suggest that higher education levels might be “protective” and associated with program adherence even without follow-up, while the impact of education level appears less pronounced when the intervention program is combined with even simple blended care, indicating that while people with lower education levels may require more support to achieve an effect, even minimal contact such as the simple EPIO_BlendedCare_ model could potentially be of benefit*.*

Lower education levels have been correlated with lower health literacy [39,76], that is, the knowledge and ability to access, understand, appraise, and use health information and services [77], which, in turn, may impact the capacity for self-management [78]. Digital and digital health literacy should therefore be considered when designing and delivering digital health interventions [79], and higher digital health literacy has, in fact, been associated with better self-management, better psychological well-being and QoL, as well as better participation in health-related decisions [80].

### Strengths and Limitations

This study had some major strengths in being able to compare findings from blended care and no-follow-up delivery models of a digital pain self-management intervention already shown to be effective in an RCT. The study also had limitations. First, the EPIO_NoFollowUp_ group was not a true part of the RCT, as recruitment for this group did not commence until after all participants had been included in the RCT. That is, only 2 of the 3 groups examined in the current study were randomized simultaneously. Comparing postintervention outcomes across groups from 2 different studies, measured at different time points, could introduce bias and interpretive challenges. There is also the issue that the EPIO_NoFollowUp_ group was recruited in 2022, whereas the RCT groups were recruited in 2019-2021. Some participants were hence involved in the study during the COVID-19 pandemic, which in itself could have impacted study comparisons. The target group, recruitment methods, and study procedures were, however, identical across studies and time points, with the same project team, study area, personnel, and locations, which could aid in minimizing potential differences in all other controllable study aspects.

Second, the number of participants in the EPIO_NoFollowUp_ group was smaller (ie, approximately half) compared with the 2 RCT groups, which may have impacted study power (ie, insufficient statistical power) and hence between-intervention comparisons related to outcomes. Potential impact of sample size on program use is, however, unlikely. Third, the number of outcomes, subscales, and time points tested in this study could potentially lead to type I errors. However, as all analyses were considered exploratory, no correction for multiple testing was made. The findings should nevertheless be interpreted with caution and corroborated by future, independent datasets. Fourth, exploratory analyses related to the potential impact of education level included small numbers, as only 51% (37/73) of the EPIO_NoFollowUp_ group completed the 12-month outcome measures (ie, compared with 99/125, 79% in the EPIO_BlendedCare_ group). Findings from these analyses should therefore also be interpreted with caution.

Fifth, there were some baseline differences between the EPIO_NoFollowUp_ group and the RCT groups related to self-reported pain conditions, including unspecified disc disorder, fibromyalgia, other types of pain, and pain duration. Pain condition or duration did, however, not appear to impact the findings in the RCT [31]. Also, the fact that these conditions were self-reported, and that participants could report more than 1 condition if they wished to do so, made identifying the potential impact of these reported differences challenging. Sixth, the poststudy interviews were conducted with participants completing the 12-month outcome measures, which may have contributed to an overrepresentation of people particularly interested in the program and program completion. The primary goal with the postintervention interviews was, however, to explore perceived experiences with the use of EPIO, including the lack of follow-up during the study, and the final sample interviewed entailed a carefully selected variation in degree of program progress and/or completion. Also, including people who had not used the EPIO program much could potentially have introduced recall bias. Finally, the majority of participants in the study were, as in most self-management interventions, female, which presents limitations for generalizability.

### Clinical Implications and Future Directions

Digital self-management interventions can be valuable support for people living with chronic pain, their health care providers, and society itself [15]. While delivery models with human support have been the most prevalent in digital health interventions so far [81], in-person guided or blended care delivery models require resources, and with health care systems under pressure [36], effective yet resource- and cost-effective interventions are clearly needed [15]. Unguided digital health interventions for chronic pain have also been shown to require lower costs, potentially being cost-effective additions to more conventional pain interventions [82].

This study implies that even user-centered, evidence-based pain self-management interventions may depend on some degree of follow-up to induce an effect. In real-world settings, mobile health apps are typically downloaded individually (eg, through the Apple App Store or Google Play Store), without additional support or follow-up. Findings also suggest that education level might impact benefit from digital interventions, particularly when delivered without follow-up. Future research should further explore these aspects, as tailoring might be essential when providing access to digital health or pain management interventions [14]. Given clinical resource limitations, providing blended care approaches to those potentially needing them most to achieve the intended effect should be explored. Some type of tailored support may also be cost-effective, and future research should ideally establish the cost-effectiveness of various levels of blended care, stratified by baseline characteristics of target users (eg, education, health literacy, and digital literacy).

Health care services under pressure further highlight the need to improve uptake, engagement, and impact of evidence-based, user-centered digital self-management interventions, without the need for pervasive health care provider involvement [83]. Taking these issues into account, factors such as access to user guidance and health information, information about program progress and feedback features, reminders and self-monitoring aspects, motivational messages, goal setting, social interaction, gamification and rewards, as well as low program cost, should further be considered essential in the process of facilitating uptake, adherence, and engagement with digital health interventions [81,84]. Findings from the current study are in line with these recommendations, as participants, for example, described having wished to participate in the study because the program was evidence-based, which created trust and interest.

### Conclusion

Digital pain self-management interventions can be of benefit to people living with chronic pain and provide important means of support for health care providers and society at large. Findings from the current study do, however, suggest that “one size does not fit all,” and when people are to engage in self-management without any form of follow-up, they might not use the intervention as much and subsequently may not benefit as intended. Methods of delivery hence emerge as essential when aiming to improve not only outreach and availability but also, essentially, use and effect. Guided interventions appear to have the greatest impact. However, education level could potentially play a role, particularly when digital interventions are delivered without guidance or follow-up. When clinical or health economy resources are limited, intervention tailoring might be necessary, with delivery with limited follow-up for some, yet blended care models with some form of guidance delivered to others.

## Appendix

Multimedia Appendix 1 EPIO_NoFollowUp_: start-up phone call template.

Multimedia Appendix 2 Guidelines and usage recommendations.

Multimedia Appendix 3 CONSORT checklist.

Multimedia Appendix 4 Sensitivity analyses comparing baseline sociodemographic characteristics for responders and nonresponders.

Multimedia Appendix 5 Comparison of EPIO_NoFollowUp_ and EPIO_BlendedCare_.

Multimedia Appendix 6 Comparison of outcomes in EPIO_NoFollowUp_ and EPIO_BlendedCare_ by education levels.

Multimedia Appendix 7 Participant education level, program module completion, and days used.

Multimedia Appendix 8 Examples of quotes related to the main themes: awareness, acceptance, and coping.

## Abbreviations

ACT: acceptance and commitment therapy
BPI: Brief Pain Inventory
CBT: cognitive behavioral therapy
CONSORT: Consolidated Standards of Reporting Trials
CPAQ-8: Chronic Pain Acceptance Questionnaire-8
ES: effect size
GLM: generalized linear model
HADS: Hospital Anxiety and Depression Scale
HRQoL: health-related quality of life
MCAR: missing completely at random
MD: mean difference
PCS: Pain Catastrophizing Scale
PVO: Hospital Privacy Protection Committee (Personvernombudet)
QoL: quality of life
RAND-36: RAND 36-Item Health Survey
RCT: randomized controlled trial
REK: Regional Committee for Medical and Health Research Ethics
SRF-18: Self-Regulatory Fatigue Scale-18
TSD: Services for Sensitive Data

## Data Availability

The datasets generated or analyzed during this study are not publicly available due to the nature of patient-sensitive information, but deidentified data are available from the corresponding author on reasonable request.
