# Supplementary material for: Delivery Model Matters for Digital Interventions in Pain Self-Management: Mixed Methods Study
Source: J Med Internet Res. 2026 Jul 20;28:e89780. doi: 10.2196/89780 (PMC13434256; doi:10.2196/89780)
Supplement: Supplementary file 2 [file jmir_v28i1e89780_app1.docx]

# **Multimedia Appendix 1**

# EPIO_NoFollowUp_: Start Up Phone Call Template

# – English version

- After completion of informed consent form and baseline measures

Thank you for the signed informed consent form and outcome measures. You received some information about the study and the program the last time we spoke on the phone (i.e., study information and screening for inclusion/exclusion criteria), but I’d like to start with some information about some of the features in the EPIO app and how to use the app you will now receive access to.

- As mentioned previously, the EPIO pain management program contains content related to psychoeducational information, coping methods, and a variety of mind-body type exercises. Hopefully this can contribute in positive ways to everyday life for you and others living with pain. There are 9 separate modules in the app, each consisting of multiple steps. The steps in each module are completed in chronological order. Modules 1-5 will also open in a fixed order, but you can then continue in the order of your choice.
- When you have completed a module, there will be a 3-day waiting period before the next module opens. This is called the practice mode. During these 3 days, we recommend that you practice the exercises to become well acquainted with the content and hence increase your potential benefit from the program before the next module opens.
- By practicing and repeating the program exercises, the program will become part of your everyday life, you will enable good health habits, and the chance of achieving effect is increased.
- You have the opportunity to make daily registrations of sleep, activity, rest, and mood. These registrations are presented in a graph under “Registration” in the app, where you can see connections and trends during your days.
- You can also create your own list of “favorites” by marking the exercises or information you like by pushing the heart button. This way, you will find it easier to retrieve your favorites.
- In settings, you have the option to set reminders to use the app. This will come as a push notification (a reminder) on your screen at your chosen time. Such reminders can be useful to set initially to remember to use the program.
- In the EPIO pain management program, you will be introduced to, among other things, relaxation and breathing exercises, and we recommend that you practice these exercises. Use of breath plays a central role in pain management, and breathing well and correctly with your diaphragm, your belly, can help relax your muscles, which again may reduce stiffness and muscle pain. Deep breathing with your belly can also help you focus, and when combined with other elements in the program, such breathing maybe even reduce attention on pain.
- The most important thing is to find something in the program that helps ***you***. Whether it's a specific type of exercise or several different ones, the important thing is to find topics or exercises you like. There are various types of exercises in the app, and some will likely feel more relevant to you than others. Some you may already be familiar with. We do however recommend that you complete the entire program to benefit the most. Repeating exercises and content will also be of great benefit.

Do you have any questions? (*If not – move on*)

- Now you can put me on speakerphone, and I will help you download the EPIO app. What kind of cellphone do you have? *(Note this on Ironkey).* Go to App Store/Google Play, search for EPIO, and download the program. The first time you start the app, you will be asked for a participant code. This is a long code containing numbers and letters. You will only need to use this one code once, so I will read the code to you *(read the participant code).* Now, you will be asked to create your own PIN code. Choose a code that is easy to remember, as we unfortunately do not have a "forgot password" button. You can always call us later if you need to retrieve the code.
- After this phone conversation**, I will send you an email** with a short guide for program use and recommendations. Reading the guide is of course voluntary, but it is an offer and something we recommend reviewing before you begin using the program. The guide can also be useful to review later.
- You have already completed a set of questionnaires as a starting point for the study. For the research study we will send you a new questionnaire after 3 months. Your questionnaire responses will help us see potential benefit from the program. You will also receive similar follow-up questions at approximately 6 and 12 months after you started the study. This will help us gather information about how things are going when you have had access to the program for a while. If circumstances prevent you from responding to one of the questionnaires packages, we would still like you to respond to those you receive later on.
- **If you experience problems with the app**, have questions, or wish to provide us with feedback, please feel free to contact the project phone or email.

Do you have any questions?

Good luck with the program and thank you for the conversation!

*REMEMBER:*

- *Be aware that everyone must have a code on their phone to use the EPIO app. If they receive a message after downloading regarding security, they must go into settings and create a lock screen on the phone. It’s wise to inform them that if they remove the code, they will also not be able to use the app.*
- *When creating a personal code, it must contain enough unique characters. 1234 is not allowed due to security. Using too many identical digits, such as 2020, is also not permitted.*
- *Note: Huawei phones may need a guideline sent via email if notifications do not to work on their phone.*
