# Supplementary material for: Delivery Model Matters for Digital Interventions in Pain Self-Management: Mixed Methods Study
Source: J Med Internet Res. 2026 Jul 20;28:e89780. doi: 10.2196/89780 (PMC13434256; doi:10.2196/89780)
Supplement: Supplementary file 3 [file jmir_v28i1e89780_app2.docx]

**Multimedia Appendix 2**

## **e-mail for EPIO_NoFollowUp_ group: - GUIDELINES AND USAGE RECOMMENDATIONS**

## **– English version**

### 1. Welcome to the pain self-management program EPIO!

Thank you for joining and helping us with this study!

The EPIO program has been created to support you who live with chronic pain, and contains information, suggestions for various methods of coping, and useful exercises you can do to support and strengthen various areas of your daily life. The program is based on scientific methods and principles that we know work, and we hope EPIO can also be of help to you. EPIO has been developed at Oslo University Hospital by researchers in close collaboration with IT developers, healthcare professionals, and people living with chronic pain.

Think of the EPIO program as a supportive tool for your day-to-day life. In a way, it can be seen as a toolbox containing ways to deal with pain. As with most activities meant to enable change and improve health and well-being though, it will take time and require effort to become well acquainted with the content and to integrate these methods into your everyday life.

When this research project is completed, the goal is for the EPIO pain self-management program to be available to everyone who wishes to use it.

### 2. Overview of the EPIO Program

EPIO has been developed based on recognized principles for effective pain self-management. The program consists of 9 modules containing information and exercises, including:

- Exercises and methods for relaxation that can help with pain
- Knowledge about planning activities and rest, the importance of self-care, enjoyable activities, and the relationship between thoughts and feelings
- Ways to reduce stress and strengthen your own stress management
- How to get better support from those around you, how you influence and can be influenced by others, and what you can do to strengthen coping in everyday life

The first five modules will be presented in a fixed order. This is to give you access to all necessary information as early as possible. Afterwards, you can choose the order to proceed, if you wish.

Some areas or modules may feel more interesting to you than others, but you will gain the most benefit from going through the entire program. Some of the content may already be familiar to you, but in that case, try to see the information as a reminder, relate it to specific exercises and routines in your everyday life, which again may make it easier to implement the strategies and make the methods a natural part of your life.

### 3. Background of the EPIO Program

In Norway, as many as one in three adults live with chronic or persistent pain at some point, making pain a serious challenge affecting many.

As you may have heard before, pain is the body's alarm signal that warns us and protects us from danger, harm, or illness. Acute pain typically arises suddenly due to injury or illness, and in this sense, pain itself is essential for survival. However, when pain lasts for a long time, such as chronic or persistent pain (>3-6 months), the "alarm signal of pain" loses its function and actually interferes more than it protects. If pain signals continuously occurs it may eventually affect so many areas of daily life that both physical and mental health can be compromised. The problem becomes not only the pain in itself but the consequences and limitations brought on by pain.

You may have already tried various treatments for pain without success. There could be many reasons for this, but the main reason is probably simply that it is incredibly difficult to get rid of pain that has lasted for a long time. Therefore, the goal of this program is not to eliminate the pain entirely, which would be very difficult, perhaps impossible, but we hope that EPIO will help you achieve less pain and gain a slightly better day-to-day life.

### 4. Using EPIO

- You will have the option to make personal registrations in EPIO. Here, you can choose whether you want to register variables such as sleep, rest, activity, and mood. If you do not choose to activate registration the first time or wish to make changes, you can return to this option later under ***"settings"***. If you choose to make such registrations, you will be asked to do this once a day, the first time you log into the app. You can also choose to postpone the registration to later in the day by pressing ***"skip"***. You will have the opportunity to see your progress in the form of a graph on ***"my page"*** and under ***"registrations"***.
- You have the opportunity to activate a daily or weekly reminder to use EPIO, known as a push notification. We recommend utilizing this at the beginning to establish continuity in use and to make using EPIO a habit in your daily life. (Please note that push notifications do not work optimally on Huawei phones, and if needed we can provide specific guidance to activate push notifications on Huawei devices).

When you enter the EPIO app, you will see a toolbar at the bottom of the screen.

- **Home button:** Shows you where you are in the program and will always take you back to the last module you worked on. The various steps in a module are arranged in a row, and you will see that they have a lock symbol attached to them. This is because you must complete the first step before the next step opens. Within the steps, you can choose whether you want to listen or read. Press the "play" button to listen to the text. The night mode is the button on the right with a crescent moon, where you can listen to the step while the screen is darkened to prevent disturbance from light. The phone will also not go into screen lock in night mode.
- **My page:** This is your personal page that provides an overview of all the modules in the program and your progression. Once you have completed a step or module, you will have the option to return to this step or module at any time.
- **Favorites:** Information or exercises you particularly enjoy can be marked as favorite through pushing the “heart” button. The step will then be added to your favorites. Over time, you will have your own easily retrievable list of favorites, where you can remove or add steps as you go.
- You must go through all the steps in each module at least once to proceed. This is important for you to gain full benefit from the program. We also recommend that you repeat the exercises several times to ensure usefulness of the program.
- After completing a module, you will enter **practice mode**. This means that you will need to wait 3 days before the next module opens. This is because we recommend practicing and repeating the exercises several times to become well-acquainted with the content and increase the usefulness of the program - before moving on to a new module. In practice mode, you will find all the steps you have gone through from the previous module open for your use. As you complete more modules, they will also become available in practice mode. You can then choose steps from your favorites or let the app select steps for you.
- If you wish to use the EPIO app without internet access, you can check **“available offline”** within each module. (Note that stable internet access is necessary to download the content of each module).
- Modules 1-5 will be presented in a fixed order. From modules 6-8, you can choose the order yourself.
- To gain full benefit from the app, it is **essential to complete all modules, preferably multiple times so that you are thoroughly familiar with the content and internalize good methods and techniques.**
- If you have questions or feedback, feel free to contact us through the study phone: *(project cell phone number listed)* (weekdays between 9:00 AM - 3:00 PM).

**Thank you for being willing to use EPIO! We hope the program will be beneficial for you.**

Best regards from the pain management team at the Department of Digital Health Research, Oslo University Hospital

*(project team names listed)*
