# Supplementary material for: Delivery Model Matters for Digital Interventions in Pain Self-Management: Mixed Methods Study
Source: J Med Internet Res. 2026 Jul 20;28:e89780. doi: 10.2196/89780 (PMC13434256; doi:10.2196/89780)
Supplement: Supplementary file 4 [file jmir_v28i1e89780_app4.docx]

**Multimedia Appendix 4.** Sensitivity analyses comparing baseline sociodemographic characteristics for responders and non-responder at 3, 6 and 12 months

|  | | **3 months** | | | **6 months** | | | **12 months** | | |
| --- | --- | --- | --- | --- | --- | --- | --- | --- | --- | --- |
|  | | **Responders**  **(n=278)** | **Non-responders**  **(n=54)** | **p-value** | **Responders**  **(n=254)** | **Non-responders**  **(n=78)** | **p-value** | **Responders**  **(n=246)** | **Non-responders**  **(n=86)** | **p-value** |
| **Age (years), median (range)** | | 49 (22-78) | 45.5 (20-81) | 0.102 | 49 (22-78) | 47.5 (20-78) | 0.277 | 49 (22-78) | 48 (20-78) | 0.579 |
| **Sex, n (%)** | |  |  | **0.020** |  |  | 0.823 |  |  | 0.477 |
|  | Female | 233 (83.8) | 38 (70.4) |  | 208 (81.9) | 63 (80.8) |  | 203 (82.5) | 68 (79.1) |  |
|  | Male | 45 (16.2) | 16 (29.6) |  | 46 (18.1) | 15 (19.2) |  | 43 (17.5) | 18 (20.9) |  |
| **Marital status, n (%)** | |  |  | 0.326 |  |  | 0.090 |  |  | **0.024** |
|  | Married/cohabiting | 189 (68.0) | 33 (61.1) |  | 176 (69.3) | 46 (59.0) |  | 173 (70.3) | 49 (57.0) |  |
|  | Single/divorced | 89 (32.0) | 21 (38.9) |  | 78 (30.7) | 32 (41.0) |  | 73 (29.7) | 37 (43.0) |  |
| **Education, n (%)** | |  |  | 0.989 |  |  | 0.677 |  |  | 0.404 |
|  | Elementary/high school | 116 (41.7) | 23 (42.6) |  | 103 (40.6) | 36 (46.1) |  | 100 (40.6) | 39 (45.3) |  |
|  | University/college ≤ 4 yrs | 111 (39.9) | 21 (38.9) |  | 103 (40.6) | 29 (37.2) |  | 103 (41.9) | 29 (33.7) |  |
|  | University/college > 4 yrs | 51 (18.3) | 10 (18.5) |  | 48 (18.9) | 13 (16.7) |  | 43 (17.5) | 18 (20.9) |  |
| **Employment, n (%)** | |  |  | 0.933 |  |  | 0.391 |  |  | 0.759 |
|  | Full-time/part time | 66 (23.7) | 13 (24.1) |  | 57 (22.4) | 22 (28.2) |  | 58 (23.6) | 21 (24.4) |  |
|  | Sick leave/disability | 195 (70.1) | 37 (68.5) |  | 179 (70.5) | 53 (67.9) |  | 171 (69.5) | 61 (70.9) |  |
|  | Retired/other | 17 (6.1) | 4 (7.4) |  | 18 (7.1) | 3 (3.8) |  | 17 (6.9) | 4 (4.7) |  |
| **100 % disability benefits, n (%)** | | 84 (30.2) | 18 (33.3) | 0.588 | 77 (30.3) | 25 (32.1) | 0.787 | 74 (30.1) | 28 (32.6) | 0.684 |
| **Income status, (EUR^a^), n (%)** | |  |  | 0.555 |  |  | 0.922 |  |  | 0.357 |
|  | < 40.000 | 55 (19.8) | 11 (20.4) |  | 50 (19.7) | 16 (20.5) |  | 46 (18.7) | 20 (23.2) |  |
|  | > 40.000 to 60.000 | 42 (15.1) | 12 (22.2) |  | 39 (15.4) | 15 (19.2) |  | 37 (15.0) | 17 (19.8) |  |
|  | > 60.000 to 80.000 | 67 (24.1) | 12 (22.2) |  | 61 (24.0) | 18 (23.1) |  | 57 (23.2) | 22 (25.6) |  |
|  | > 80.000 to 100.000 | 56 (20.1) | 12 (22.2) |  | 54 (21.3) | 14 (18.0) |  | 56 (22.8) | 12 (14.0) |  |
|  | > 100.000 | 58 (20.9) | 7 (13.0) |  | 50 (19.7) | 15 (19.2) |  | 50 (20.3) | 15 (17.4) |  |
| **Years living with pain, n (%)** | |  |  | 0.341 |  |  | 0.579 |  |  | 0.994 |
|  | ≤ 10 years | 108 (38.8) | 23 (42.6) |  | 106 (41.7) | 25 (32.2) |  | 96 (39.0) | 35 (40.7) |  |
|  | > 10 years | 170 (61.2) | 31 (57.4) |  | 148 (58.3) | 53 (67.9) |  | 150 (61.0) | 51 (59.3) |  |

^a^EUR = 1 EURO is approximately 1.16 USD and approximately 10,98 Norwegian kroner (Summer 2026)
