# Supplementary material for: Delivery Model Matters for Digital Interventions in Pain Self-Management: Mixed Methods Study
Source: J Med Internet Res. 2026 Jul 20;28:e89780. doi: 10.2196/89780 (PMC13434256; doi:10.2196/89780)
Supplement: Supplementary file 5 [file jmir_v28i1e89780_app5.docx]

**Multimedia Appendix 5**

Comparison EPIO_NoFollowUp_ and EPIO_BlendedCare_ groups at baseline, 3, 6 and 12 months. Estimated marginal means from generalized linear mixed models.

|  | | EPIO_NoFollowUp_  n=73 | | EPIO_BlendedCare_  n=125 | | Between-group differences | | |
| --- | --- | --- | --- | --- | --- | --- | --- | --- |
|  | | **M** | **[95% CI]** | **M** | **(95% CI)** | **MD** | **(95% CI)** | *P-value* |
| Pain interference (BPI^a^)^b^ | |  |  |  |  |  |  |  |
|  | Baseline | 5.0 | [4.5 to 5.4] | 4.9 | [4.6 to 5.3] |  |  |  |
|  | 3 months | 5.1 | [4.5 to 5.7] | 4.5 | [4.1 to 4.8] | 0.5 | [-0.1 to 1.1] | .080 |
|  | 6 months | 4.4 | [3.8 to 5.0] | 4.5 | [4.1 to 4.9] | -0.2 | [-0.8 to 0.5] | .614 |
|  | 12 months | 4.4 | [3.8 to 5.1] | 4.4 | [4.0 to 4.9] | -0.1 | [-0.9 to 0.7] | .805 |
| Pain severity (BPI^a^)^c^ | |  |  |  |  |  |  |  |
|  | Baseline | 5.0 | [4.7 to 5.4] | 5.1 | [4.8 to 5.4] |  |  |  |
|  | 3 months | 5.0 | [4.6 to 5.4] | 4.9 | [4.6 to 5.2] | 0.2 | [-0.2 to 0.6] | .394 |
|  | 6 months | 4.6 | [4.1 to 5.1] | 4.9 | [4.6 to 5.2] | -0.2 | [-0.7 to 0.3] | .413 |
|  | 12 months | 4.9 | [4.4 to 5.3] | 4.7 | [4.4 to 5.1] | 0.2 | [-0.3 to 0.8] | .380 |
| Anxiety (HADS-A^d^) | |  |  |  |  |  |  |  |
|  | Baseline | 8.1 | [7.2 to 9.0] | 7.2 | [6.5 to 7.9] |  |  |  |
|  | 3 months | 7.4 | [6.5 to 8.3] | 6.4 | [5.7 to 7.1] | 0.0 | [-1.0 to 1.1] | .923 |
|  | 6 months | 7.6 | [6.5 to 8.8] | 6.4 | [5.7 to 7.1] | 0.3 | [-1.0 to 1.6] | .651 |
|  | 12 months | 7.8 | [6.9 to 8.8] | 6.1 | [5.4 to 6.9] | 0.8 | [-0.4 to 2.0] | .196 |
| Depression (HADS-D^e^) | |  |  |  |  |  |  |  |
|  | Baseline | 7.3 | [6.5 to 8.1] | 6.9 | [6.1 to 7.6] |  |  |  |
|  | 3 months | 6.7 | [5.7 to 7.6] | 5.6 | [5.0 to 6.2] | 0.6 | [-0.3 to 1.6] | .203 |
|  | 6 months | 5.8 | [4.8 to 6.8] | 5.7 | [5.1 to 6.4] | -0.4 | [-1.4 to 0.6] | .477 |
|  | 12 months | 6.7 | [5.7 to 7.7] | 5.5 | [4.8 to 6.2] | 0.7 | [-0.4 to 1.8] | .188 |
| Self-Regulatory Fatigue (SRF-18^f^) | |  |  |  |  |  |  |  |
|  | Baseline | 55.6 | [52.6 t0 58.2] | 54.1 | [52.3 to 55.9] |  |  |  |
|  | 3 months | 55.4 | [52.6 to 58.2] | 51.0 | [49.1 to 52.8] | 2.9 | [0.3 to 5.4] | .026 |
|  | 6 months | 54.1 | [51.2 to 57.0] | 52.7 | [50.7 to 54.7] | -0.1 | [-3.0 to 2.7] | .935 |
|  | 12 months | 54.7 | [51.5 to 57.9] | 51.0 | [49.0 to 53.1] | 2.1 | [-1.4 to 5.6] | .237 |
| HRQoL^g^ (RAND-36^h^) | |  |  |  |  |  |  |  |
| *Physical functioning* | |  |  |  |  |  |  |  |
|  | Baseline | 57.9 | [52.9 to 63.0] | 56.8 | [52.3 to 61.2] |  |  |  |
|  | 3 months | 60.5 | [54.9 to 66.2] | 61.3 | [56.7 to 65.9] | -1.9 | [-6.1 to 2.2] | .362 |
|  | 6 months | 64.1 | [58.5 to 69.6] | 61.4 | [56.8 to 66.0] | 1.5 | [-3.2 to 6.1] | .536 |
|  | 12-months | 63.2 | [56.7 to 69.8] | 60.4 | [55.7 to 65.2] | 1.6 | [-3.6 to 6.8] | .544 |
| *Role-Physical* | |  |  |  |  |  |  |  |
|  | Baseline | 18.5 | [11.2 to 25.8] | 19.6 | [14.1 to 25.1] |  |  |  |
|  | 3 months | 26.7 | [17.8 to 35.6] | 25.3 | [19.0 to 31.6] | 2.5 | [-7.8 to 12.7] | .634 |
|  | 6 months | 31.1 | [20.6 to 41.5] | 25.9 | [19.3 to 32.6] | 6.2 | [-5.1 to 17.6] | .282 |
|  | 12 months | 27.4 | [17.4 to 37.4] | 26.3 | [19.4 to 33.2] | 2.2 | [-9.2 to 13.5] | .707 |
| *Bodily pain* | |  |  |  |  |  |  |  |
|  | Baseline | 26.2 | [22.3 to 30.2] | 27.5 | [24.8 to 30.1] |  |  |  |
|  | 3 months | 31.9 | [26.5 to 37.4] | 30.7 | [27.3 to 34.2] | 2.4 | [-3.8 to 8.7] | .448 |
|  | 6 months | 35.7 | [30.3 to 41.0] | 32.9 | [29.5 to 36.3] | 4.0 | [-1.4 to 9.5] | .148 |
|  | 12 months | 34.0 | [28.3 to 39.7] | 35.1 | [31.0 to 39.2] | 0.1 | [-6.4 to 6.7] | .965 |
| General health | |  |  |  |  |  |  |  |
|  | Baseline | 37.7 | [33.1 to 42.3] | 37.1 | [33.6 to 40.5] |  |  |  |
|  | 3 months | 38.5 | [33.2 to 43.8] | 42.0 | [38.5 to 45.5] | -4.1 | [-9.1 to 1.0] | .115 |
|  | 6 months | 44.8 | [40.1 to 49.4] | 40.9 | [37.3 to 44.6] | 3.2 | [-1.9 to 8.3] | .215 |
|  | 12 months | 41.3 | [35.9 to 46.6] | 41.6 | [37.8 to 45.3] | -0.9 | [-6.6 to 4.8] | .756 |
| *Vitality* | |  |  |  |  |  |  |  |
|  | Baseline | 24.4 | [20.1 to 28.7] | 26.9 | [23.6 to 30.2] |  |  |  |
|  | 3 months | 30.4 | [24.7 to 36.2] | 33.6 | [29.8 to 37.4] | -0.6 | [-6.6 to 5.4] | .839 |
|  | 6 months | 30.9 | [25.7 to 36.1] | 29.9 | [26.1 to 33.8] | 3.5 | [-2.4 to 9.4] | .246 |
|  | 12 months | 25.1 | [19.5 to 30.8] | 33.0 | [28.7 to 37.4] | -5.4 | [-11.7 to 0.9] | .096 |
| *Social functioning* | |  |  |  |  |  |  |  |
|  | Baseline | 45.4 | [39.7 to 51.1] | 46.7 | [42.3 to 51.1] |  |  |  |
|  | 3 months | 48.8 | [42.4 to 55.1] | 53.8 | [49.2 to 58.5] | -3.7 | [-10.8 to 3.3] | .299 |
|  | 6 months | 53.2 | [46.3 to 60.1] | 56.2 | [51.2 to 61.2] | -1.7 | [-10.8 to 3.3] | .683 |
|  | 12 months | 52.9 | [45.3 to 60.6] | 55.6 | [50.6 to 60.7] | -1.4 | [-10.1 to 7.3] | .755 |
| *Role-Emotional* | |  |  |  |  |  |  |  |
|  | Baseline | 53.4 | [43.5 to 63.4] | 48.5 | [40.7 to 56.3] |  |  |  |
|  | 3 months | 56.1 | [45.3 to 66.9] | 57.8 | [49.8 to 65.8] | -6.6 | [-22.0 to 8.8] | .402 |
|  | 6 months | 51.3 | [39.4 to 63.2] | 52.5 | [44.0 to 61.1] | -6.1 | [-21.4 to 9.1] | .431 |
|  | 12 months | 51.3 | [37.3 to 65.4] | 62.2 | [53.4 to 70.9] | -15.8 | [-35.3 to 3.7] | .113 |
| *Mental health* | |  |  |  |  |  |  |  |
|  | Baseline | 62.5 | [58.6 to 66.3] | 65.9 | [62.7 to 69.1] |  |  |  |
|  | 3 months | 64.0 | [59.7 to 68.2] | 68.4 | [64.9 to 71.9] | -1.0 | [-5.9 to 3.8] | .679 |
|  | 6 months | 65.0 | [60.0 to 70.0] | 65.6 | [62.2 to 69.0] | 2.8 | [-3.7 to 9.4] | .394 |
|  | 12 months | 63.7 | [58.6 to 68.8] | 68.0 | [64.4 to 71.7] | -0.9 | [-6.9 to 5.1] | .766 |
| Pain catastrophizing (PCS^i^) | |  |  |  |  |  |  |  |
| *Rumination* | |  |  |  |  |  |  |  |
|  | Baseline | 7.6 | [6.7 to 8.6] | 7.2 | [6.6 to 7.9] |  |  |  |
|  | 3 months | 6.7 | [5.7 to 7.6] | 6.4 | [5.7 to 7.1] | -0.2 | [-1.2 to 0.9] | .770 |
|  | 6 months | 6.6 | [5.5 to 7.8] | 6.0 | [5.3 to 6.7] | 0.2 | [-1.0 to 1.5] | .733 |
|  | 12 months | 6.2 | [5.0 to 7.4] | 5.3 | [4.6 to 6.0] | 0.5 | [-0.9 to 1.9] | .487 |
| *Magnification* | |  |  |  |  |  |  |  |
|  | Baseline | 3.8 | [3.2 to 4.3] | 3.3 | [2.9 to 3.7] |  |  |  |
|  | 3 months | 3.2 | [2.6 to 3.8] | 3.0 | [2.6 to 3.4] | -0.2 | [-0.9 to 0.5] | .560 |
|  | 6 months | 3.4 | [2.8 to 4.1] | 2.9 | [2.4 to 3.3] | 0.1 | [-0.6 to 0.8] | .748 |
|  | 12 months | 3.0 | [2.3 to 3.6] | 2.5 | [2.1 to 3.0] | 0.0 | [-0.9 to 0.8] | .931 |
| *Helplessness* | |  |  |  |  |  |  |  |
|  | Baseline | 9.9 | [8.6 to 11.1] | 8.2 | [7.3 to 9.0] |  |  |  |
|  | 3 months | 8.5 | [7.1 to 9.8] | 7.8 | [6.9 to 8.7] | -1.0 | [-2.5 to 0.5] | .211 |
|  | 6 months | 7.4 | [5.9 to 8.9] | 7.3 | [6.5 to 8.2] | -1.6 | [-3.3 to 0.1] | .062 |
|  | 12 months | 8.3 | [6.8 to 9.8] | 6.6 | [5.7 to 7.5] | 0.0 | [-1.8 to 1.8] | .989 |
| *PCS total* | |  |  |  |  |  |  |  |
|  | Baseline | 21.3 | [18.7 to 23.8] | 18.7 | [17.0 to 20.4] |  |  |  |
|  | 3 months | 18.3 | [15.8 to 20.9] | 17.2 | [15.4 to 18.9] | -1.4 | [-4.0 to 1.2] | .297 |
|  | 6 months | 17.4 | [14.5 to 20.3] | 16.2 | [14.4 to 17.9] | -1.3 | [-4.5 to 1.8] | .400 |
|  | 12 months | 17.5 | [14.5 to 20.5] | 14.5 | [12.6 to 16.4] | 0.4 | [-3.1 to 4.0] | .816 |
| Chronic pain acceptance (CPAQ^j^) | |  |  |  |  |  |  |  |
| *Willingness* | |  |  |  |  |  |  |  |
|  | Baseline | 13.4 | [12.8 to 14.0] | 13.6 | [13.1 to 14.1] |  |  |  |
|  | 3 months | 14.1 | [13.6 to 14.7] | 13.8 | [13.2 to 14.3] | 0.6 | [-0.3 to 1.4] | .189 |
|  | 6 months | 14.2 | [13.5 to 14.9] | 13.6 | [13.0 to 14.1] | 0.8 | [-0.1 to 1.8] | .089 |
|  | 12 months | 14.7 | [13.6 to 15.7] | 13.8 | [13.3 to 14.4] | 1.0 | [-0.3 to 2.3] | .128 |
| *Activity engagement* | |  |  |  |  |  |  |  |
|  | Baseline | 13.7 | [13.0 to 14.5] | 13.6 | [12.9 to 14.3] |  |  |  |
|  | 3 months | 14.6 | [13.8 to 15.4] | 14.2 | [13.6 to 14.8] | 0.3 | [-0.6 to 1.2] | .495 |
|  | 6 months | 14.9 | [14.1 to 15.7] | 13.8 | [13.2 to 14.4] | 1.0 | [-0.1 to 2.1] | .087 |
|  | 12 months | 14.0 | [13.1 to 14.9] | 14.6 | [13.9 to 15.2] | -0.7 | [-1.7 to 0.4] | .214 |
| *CPAQ total* | |  |  |  |  |  |  |  |
|  | Baseline | 27.1 | [26.0 to 28.3] | 27.2 | [26.1 to 28.3] |  |  |  |
|  | 3 months | 28.8 | [27.7 to 29.9] | 28.0 | [27.1 to 28.9] | 0.9 | [-0.6 to 2.4] | .225 |
|  | 6 months | 29.1 | [27.8 to 30.4] | 27.4 | [26.4 to 28.4] | 1.8 | [-0.03 to 3.6] | .054 |
|  | 12 months | 28.6 | [27.2 to 30.1] | 28.4 | [27.4 to 29.4] | 0.3 | [-1.6 to 2.3] | .738 |

^a^BPI: Brief Pain Inventory.

^b^Subscale of the Brief Pain Inventory (score range 0-10; a higher score indicates higher interference in life).

^c^Subscale of the Brief Pain Inventory (score range 0-10; a higher score indicates higher severity).

^d^HADS-A: Hospital Anxiety and Depression Scale-Anxiety subscale (score range 0-21; a higher score indicates a higher degree of anxiety).

^e^HADS-D: Hospital Anxiety and Depression Scale-Depression subscale (score range 0-21; a higher score indicates a higher degree of depression).

^f^SRF-18: Self-regulatory Fatigue 18 scale (score range 18-90; a higher score indicates higher self-regulatory fatigue).

^g^HRQoL: health-related quality of life.

^h^RAND-36: RAND 36-Item scale (score range 0-100; a higher score indicates higher emotional well-being).

^i^PCS: Pain Catastrophizing Scale (score range 0-52; a higher score indicates higher catastrophizing).

^j^CPAQ: Chronic Pain Acceptance Questionnaire (score range 0-52; a higher score indicates a higher acceptance of pain).

Abbreviations: M, mean; MD, mean difference; CI, confidence interval
