# Supplementary material for: Delivery Model Matters for Digital Interventions in Pain Self-Management: Mixed Methods Study
Source: J Med Internet Res. 2026 Jul 20;28:e89780. doi: 10.2196/89780 (PMC13434256; doi:10.2196/89780)
Supplement: Supplementary file 6 [file jmir_v28i1e89780_app6.docx]

**Multimedia Appendix 6**

Comparison of outcomes in EPIO_NoFollowUp_ and EPIO_BlendedCare_ by education levels at baseline, 3, 6 and 12 months. Estimated marginal means from generalized linear mixed models.

|  | | | EPIO_NoFollowUp_ | | | | | | | | EPIO_BlendedCare_ | | | | | | |
| --- | --- | --- | --- | --- | --- | --- | --- | --- | --- | --- | --- | --- | --- | --- | --- | --- | --- |
|  | | |  | | | Between-group differences | | | | |  | | | | Between-group differences | | |
|  | | | **n** | **M** | **[95% CI]** | **B** | **[95% CI]** | | ***P-value*** | | **n** | | **M** | **[95%CI]** | **B** | **[95% CI]** | *P-value* |
| Pain interference (BPI^a^)^b^ | | |  |  |  |  |  | |  | |  | |  |  |  |  |  |
|  | Baseline | |  |  |  |  |  | |  | |  | |  |  |  |  |  |
|  |  | Elementary/high school | 31 | 5.4 | [4.7 to 6.2] |  |  | |  | | 49 | | 5.4 | [4.8 to 6.0] |  |  |  |
|  |  | University/college ≤ 4 years | 28 | 4.8 | [4.1 to 5.6] |  |  | |  | | 55 | | 4.4 | [3.9 to 5.0] |  |  |  |
|  |  | University/college > 4 years | 14 | 4.3 | [3.2 to 5.4] |  |  | |  | | 21 | | 4.9 | [3.6 to 4.8] |  |  |  |
|  | 3 months | |  |  |  |  |  | |  | |  | |  |  |  |  |  |
|  |  | Elementary/high school | 31 | 5.8 | [5.0 to 6.7] |  |  | |  | | 49 | | 4.9 | [4.3 to 5.2] |  |  |  |
|  |  | University/college ≤ 4 years | 28 | 5.0 | [4.2 to 5.9] | -0.2 | [-1.3 to 0.9] | | .724 | | 55 | | 4.1 | [3.6 to 4.8] | 0.3 | [-0.5 to 1.0] | .486 |
|  |  | University/college > 4 years | 14 | 3.6 | [2.3 to 4.8] | -1.1 | [-2.6 to 0.3] | | .122 | | 21 | | 4.4 | [3.4 to 5.3] | 0.0 | [-1.0 to 1.0] | .986 |
|  | 6 months | |  |  |  |  |  | |  | |  | |  |  |  |  |  |
|  |  | Elementary/high school | 31 | 5.1 | [4.2 to 6.0] |  |  | |  | | 49 | | 5.1 | [4.5 to 5.8] |  |  |  |
|  |  | University/college ≤ 4 years | 28 | 4.3 | [3.4 to 5.2] | -0.2 | [-1.4 to 1.0] | | .725 | | 55 | | 4.1 | [3.5 to 4.7] | 0.0 | [-0.8 to 0.8] | .977 |
|  |  | University/college > 4 years | 14 | 3.2 | [2.0 to 4.5] | -0.8 | [-2.3 to 0.7] | | .289 | | 21 | | 4.1 | [3.2 to 5.1] | -0.5 | [-1.5 to 0.6] | .420 |
|  | 12 months | |  |  |  |  |  | |  | |  | |  |  |  |  |  |
|  |  | Elementary/high school | 31 | 5.4 | [4.3 to 6.5] |  |  | |  | | 49 | | 4.8 | [4.2 to 5.5] |  |  |  |
|  |  | University/college ≤ 4 years | 28 | 4.1 | [3.2 to 5.0] | -0.7 | [-2.0 to 0.6] | | .309 | | 55 | | 4.1 | [3.6 to 4.7] | 0.3 | [-0.5 to 1.1] | .475 |
|  |  | University/college > 4 years | 14 | 3.4 | [2.1 to 4.6] | -0.9 | [-2.5 to 0.6] | | .244 | | 21 | | 4.2 | [3.3 to 5.2] | 0.0 | [-1.1 to 1.0] | .945 |
| Pain severity (BPI^a^)^c^ | | |  |  |  |  |  | |  | |  | |  |  |  |  |  |
|  | Baseline | |  |  |  |  |  | |  | |  | |  |  |  |  |  |
|  |  | Elementary/high school | 31 | 5.4 | [4.9 to 5.9] |  |  | |  | | 49 | | 5.5 | [5.1 to 6.0] |  |  |  |
|  |  | University/college ≤ 4 years | 28 | 4.6 | [4.1 to 5.2] |  |  | |  | | 55 | | 5.1 | [4.6 to 5.5] |  |  |  |
|  |  | University/college > 4 years | 14 | 4.9 | [4.2 to 5.7] |  |  | |  | | 21 | | 4.3 | [3.6 to 5.0] |  |  |  |
|  | 3 months | |  |  |  |  |  | |  | |  | |  |  |  |  |  |
|  |  | Elementary/high school | 31 | 5.6 | [5.0 to 6.2] |  |  | |  | | 49 | | 5.4 | [5.0 to 5.9] |  |  |  |
|  |  | University/college ≤ 4 years | 28 | 4.9 | [4.3 to 5.4] | 0.1 | [-0.8 to 0.9] | | .870 | | 55 | | 4.7 | [4.2 to 5.1] | -0.3 | [-0.9 to 0.2] | .232 |
|  |  | University/college > 4 years | 14 | 4.0 | [3.1 to 4.9] | -1.1 | [-2.1 to 0.0] | | **.041** | | 21 | | 4.1 | [3.3 to 4.8] | -0.2 | [-0.9 to 0.5] | .575 |
|  | 6 months | |  |  |  |  |  | |  | |  | |  |  |  |  |  |
|  |  | Elementary/high school | 31 | 5.5 | [4.8 to 6.1] |  |  | |  | | 49 | | 5.5 | [5.0 to 6.0] |  |  |  |
|  |  | University/college ≤ 4 years | 28 | 4.3 | [3.7 to 4.9] | -0.4 | [-1.3 to 0.5] | | .366 | | 55 | | 4.6 | [4.1 to 5.0] | -0.5 | [-1.1 to 0.1] | .081 |
|  |  | University/college > 4 years | 14 | 3.5 | [2.6 to 4.4] | -1.5 | [-2.6 to -0.5] | | **.005** | | 21 | | 4.4 | [3.7 to 5.1] | 0.1 | [-0.7 to 0.8] | .844 |
|  | 12 months | |  |  |  |  |  | |  | |  | |  |  |  |  |  |
|  |  | Elementary/high school | 31 | 5.5 | [4.7 to 6.3] |  |  | |  | | 49 | | 5.2 | [4.7 to 5.7] |  |  |  |
|  |  | University/college ≤ 4 years | 28 | 4.7 | [4.1 to 5.4] | 0.0 | [-0.9 to 1.0] | | .951 | | 55 | | 4.5 | [4.0 to 4.9] | -0.3 | [-0.8 to 0.3] | .370 |
|  |  | University/college > 4 years | 14 | 4.0 | [3.1 to 4.9] | -1.0 | [-2.2 to 0.2] | | .091 | | 21 | | 4.3 | [3.5 to 5.0] | 0.3 | [-0.5 to 1.0] | .496 |
| Anxiety (HADS-A^d^) | | |  |  |  |  |  | |  | |  | |  |  |  |  |  |
|  | Baseline | |  |  |  |  |  | |  | |  | |  |  |  |  |  |
|  |  | Elementary/high school | 31 | 8.5 | [7.3 to 9.8] |  |  | |  | | 49 | | 7.9 | [6.8 to 9.0] |  |  |  |
|  |  | University/college ≤ 4 years | 28 | 7.8 | [6.5 to 9.1] |  |  | |  | | 55 | | 6.5 | [5.5 to 7.6] |  |  |  |
|  |  | University/college > 4 years | 14 | 7.7 | [5.8 to 9.6] |  |  | |  | | 21 | | 7.1 | [5.4 to 8.8] |  |  |  |
|  | 3 months | |  |  |  |  |  | |  | |  | |  |  |  |  |  |
|  |  | Elementary/high school | 31 | 7.9 | [6.4 to 9.5] |  |  | |  | | 49 | | 7.2 | [6.0 to 8.3] |  |  |  |
|  |  | University/college ≤ 4 years | 28 | 7.5 | [6.0 to 9.0] | 0,3 | [-1.6 to 2.3] | | .744 | | 55 | | 6.0 | [5.0 to 7.1] | 0.2 | [-1.0 to 1.4] | .710 |
|  |  | University/college > 4 years | 14 | 6.1 | [3.9 to 8.3] | -1.0 | [-3.4 to 1.5] | | .440 | | 21 | | 5.9 | [4.2 to 7.6] | -0.4 | [-2.0 to 1.2] | .608 |
|  | 6 months | |  |  |  |  |  | |  | |  | |  |  |  |  |  |
|  |  | Elementary/high school | 31 | 9.2 | [7.6 to 10.8] |  |  | |  | | 49 | | 7.2 | [6.0 to 8.4] |  |  |  |
|  |  | University/college ≤ 4 years | 28 | 7.4 | [5.8 to 8.9] | -1.1 | [-3.1 to 1.0] | | .312 | | 55 | | 5.9 | [4.8 to 7.0] | 0.0 | [-1.2 to 1.3] | .939 |
|  |  | University/college > 4 years | 14 | 5.3 | [3.1 to 7.5] | -3.1 | [-5.6 to -0.6] | | **.017** | | 21 | | 6.1 | [4.4 to 7.8] | -0.3 | [-1.9 to 1.3] | .722 |
|  | 12 months | |  |  |  |  |  | |  | |  | |  |  |  |  |  |
|  |  | Elementary/high school | 31 | 9.3 | [7.4 to 11.2] |  |  | |  | | 49 | | 6.9 | [5.7 to 8.1] |  |  |  |
|  |  | University/college ≤ 4 years | 28 | 7.4 | [5.9 to 9.0] | -1.1 | [-3.4 to 1.2] | | .331 | | 55 | | 5.8 | [4.7 to 6.9] | 0.2 | [-1.0 to 1.5] | .695 |
|  |  | University/college > 4 years | 14 | 6.4 | [4.2 to 8.6] | -2.2 | [-4.8 to 0.6] | | .129 | | 21 | | 5.3 | [3.5 to 7.0] | 0.8 | [-2.5 to 0.8] | .332 |
|  |  |  |  |  |  |  |  | |  | |  | |  |  |  |  |  |
| Depression (HADS-D^e^) | | |  |  |  |  |  | |  | |  | |  |  |  |  |  |
|  | Baseline | |  |  |  |  |  | |  | |  | |  |  |  |  |  |
|  |  | Elementary/high school | 31 | 7.4 | [6.1 to 8.6] |  |  | |  | | 49 | | 7.7 | [6.7 to 8.7] |  |  |  |
|  |  | University/college ≤ 4 years | 28 | 7.1 | [5.8 to 8.4] |  |  | |  | | 55 | | 6.3 | [5.3 to 7.3] |  |  |  |
|  |  | University/college > 4 years | 14 | 7.4 | [5.6 to 9.3] |  |  | |  | | 21 | | 6.4 | [4.9 to 8.0] |  |  |  |
|  | 3 months | |  |  |  |  |  | |  | |  | |  |  |  |  |  |
|  |  | Elementary/high school | 31 | 6.8 | [5.4 to 8.3] |  |  | |  | | 49 | | 6.3 | [5.3 to 7.4] |  |  |  |
|  |  | University/college ≤ 4 years | 28 | 6.8 | [5.4 to 8.2] | 0.3 | [-1.5 to 2.0] | | .770 | | 55 | | 5.4 | [4.4 to 6.4] | 0.4 | [-0.7 to 1.6] | .452 |
|  |  | University/college > 4 years | 14 | 6.0 | [3.9 to 8.1] | -0.9 | [-3.1 to 1.3] | | .408 | | 21 | | 4.4 | [2.8 to 6.0] | -0.7 | [-2.1 to 0.8] | .369 |
|  | 6 months | |  |  |  |  |  | |  | |  | |  |  |  |  |  |
|  |  | Elementary/high school | 31 | 5.9 | [4.4 to 7.5] |  |  | |  | | 49 | | 6.4 | [5.3 to 7.5] |  |  |  |
|  |  | University/college ≤ 4 years | 28 | 5.9 | [4.4 to 7.4] | 0.3 | [-1.6 to 2.1] | | .786 | | 55 | | 5.5 | [4.4 to 6.5] | 0.5 | [-0.7 to 1.6] | .449 |
|  |  | University/college > 4 years | 14 | 5.3 | [3.2 to 7.4] | -0.7 | [-2.9 to 1.5] | | .542 | | 21 | | 5.0 | [3.4 to 6.6] | -0.2 | [-1.7 to 1.4] | .840 |
|  | 12 months | |  |  |  |  |  | |  | |  | |  |  |  |  |  |
|  |  | Elementary/high school | 31 | 7.6 | [5.8 to 9.4] |  |  | |  | | 49 | | 6.3 | [5.2 to 7.4] |  |  |  |
|  |  | University/college ≤ 4 years | 28 | 6.5 | [5.0 to 8.0] | -0.8 | [-2.8 to 1.2] | | .441 | | 55 | | 5.3 | [4.2 to 6.3] | 0.3 | [-0.8 to 1.5] | .572 |
|  |  | University/college > 4 years | 14 | 5.8 | [3.7 to 7.9] | -1.8 | [-4.2 to 0.6] | | .141 | | 21 | | 4.4 | [2.8 to 6.1] | -0.6 | [-2.2 to 0.9] | .425 |
| Self-Regulatory Fatigue (SRF-18^f^) | | |  |  |  |  |  | |  | |  | |  |  |  |  |  |
|  | Baseline | |  |  |  |  |  | |  | |  | |  |  |  |  |  |
|  |  | Elementary/high school | 31 | 58.6 | [55.1 to 62.0] |  |  | |  | | 49 | | 57.2 | [54.3 to 60.0] |  |  |  |
|  |  | University/college ≤ 4 years | 28 | 54.9 | [51.2 to 58.5] |  |  | |  | | 55 | | 53.3 | [50.5 to 56.0] |  |  |  |
|  |  | University/college > 4 years | 14 | 50.6 | [45.5 to 55.8] |  |  | |  | | 21 | | 49.1 | [44.8 to 53.5] |  |  |  |
|  | 3 months | |  |  |  |  |  | |  | |  | |  |  |  |  |  |
|  |  | Elementary/high school | 31 | 57.8 | [53.8 to 61.8] |  |  | |  | | 49 | | 53.9 | [51.0 to 56.9] |  |  |  |
|  |  | University/college ≤ 4 years | 28 | 56.5 | [52.6 to 60.4] | 2.4 | [-2.5 to 7-3] | | .330 | | 55 | | 49.1 | [46.3 to 51.9] | -0.9 | [-4.0 to 2.2] | .566 |
|  |  | University/college > 4 years | 14 | 47.8 | [42.0 to 53.6] | -2.1 | [-8.3 to 4.1] | | .516 | | 21 | | 49.0 | [44.5 to 53.5] | 3.1 | [-1.0 to 7.2] | .137 |
|  | 6 months | |  |  |  |  |  | |  | |  | |  |  |  |  |  |
|  |  | Elementary/high school | 31 | 57.3 | [53.1 to 61.5] |  |  | |  | | 49 | | 54.5 | [51.4 to 57.6] |  |  |  |
|  |  | University/college ≤ 4 years | 28 | 54.6 | [50.5 to 58.7] | 1.1 | [-4.2 to 6.3] | | .690 | | 55 | | 53.0 | [50.2 to 55.9] | 2.5 | [-0.8 to 5.7] | .139 |
|  |  | University/college > 4 years | 14 | 46.5 | [40.7 to 52.4] | -2.1 | [-8.3 to 4.1] | | .516 | | 21 | | 47.4 | [42.9 to 51.9] | 0.9 | [-3.3 to 5.2] | .622 |
|  | 12 months | |  |  |  |  |  | |  | |  | |  |  |  |  |  |
|  |  | Elementary/high school | 31 | 58.4 | [53.5 to 63.3] |  |  | |  | | 49 | | 53.7 | [50.6 to 56.7] |  |  |  |
|  |  | University/college ≤ 4 years | 28 | 54.4 | [50.3 to 58.5] | -0.3 | [-6.1 to 5.5] | | .915 | | 55 | | 50.6 | [47.8 to 53.4] | 0.8 | [-2.4 to 4.1] | .612 |
|  |  | University/college > 4 years | 14 | 48.7 | [42.8 to 54.5] | -1.9 | [-8.7 to 5.0] | | .594 | | 21 | | 46.0 | [41.4 to 50.7] | 0.4 | [-3.9 to 4.7] | .855 |
| HRQoL^g^ (RAND-36^h^) | | |  |  |  |  |  | |  | |  | |  |  |  |  |  |
| *Physical functioning* | | |  |  |  |  |  | |  | |  | |  |  |  |  |  |
|  | Baseline | |  |  |  |  |  | |  | |  | |  |  |  |  |  |
|  |  | Elementary/high school | 31 | 54.0 | [46.2 to 61.8] |  |  | |  | | 49 | | 45.5 | [38.8 to 52.2] |  |  |  |
|  |  | University/college ≤ 4 years | 28 | 59.5 | [51.3 to 67.7] |  |  | |  | | 55 | | 65.8 | [59.5 to 72.1] |  |  |  |
|  |  | University/college > 4 years | 14 | 63.6 | [52.0 to 75.2] |  |  | |  | | 21 | | 59.3 | [49.0 to 69.5] |  |  |  |
|  | 3 months | |  |  |  |  |  | |  | |  | |  |  |  |  |  |
|  |  | Elementary/high school | 31 | 53.0 | [44.5 to 61.5] |  |  | |  | | 49 | | 51.6 | [44.7 to 58.5] |  |  |  |
|  |  | University/college ≤ 4 years | 28 | 63.2 | [54.6 to 71.8] | 4.8 | [-2.9 to 12.5] | | .224 | | 55 | | 67.0 | [60.5 to 73.4] | -5.0 | [-11.0 to 1.0] | .102 |
|  |  | University/college > 4 years | 14 | 70.5 | [58.1 to 82.9] | 7.9 | [-1.7 to 17.7] | | .110 | | 21 | | 68.8 | [58.4 to 79.2] | 3.4 | [-4.5 to 11.2] | .398 |
|  | 6 months | |  |  |  |  |  | |  | |  | |  |  |  |  |  |
|  |  | Elementary/high school | 31 | 58.2 | [49.4 to 66.9] |  |  | |  | | 49 | | 50.1 | [43.1 to 57.2] |  |  |  |
|  |  | University/college ≤ 4 years | 28 | 63.8 | [55.0 to 72.6] | 0.2 | [-8.0 to 8.4] | | .960 | | 55 | | 69.0 | [62.5 to 75.6] | -1.4 | [-7.7 to 4.8] | .653 |
|  |  | University/college > 4 years | 14 | 76.4 | [63.9 to 88.8] | 8.7 | [-1.3 to 18.6] | | .090 | | 21 | | 67.3 | [56.8 to 77.8] | 3.3 | [-4.8 to 11.4] | .419 |
|  | 12 months | |  |  |  |  |  | |  | |  | |  |  |  |  |  |
|  |  | Elementary/high school | 31 | 56.9 | [47.3 to 66.5] |  |  | |  | | 49 | | 49.9 | [42.9 to 56.9] |  |  |  |
|  |  | University/college ≤ 4 years | 28 | 66.1 | [57.3 to 74.9] | 3.7 | [-5.3 to 12.8] | | .421 | | 55 | | 67.4 | [60.9 to 74.0] | -2.8 | [-9.0 to 3.4] | .377 |
|  |  | University/college > 4 years | 14 | 69.7 | [57.2 to 82.1] | 3.2 | [-7.5 to 14.0] | | .556 | | 21 | | 66.5 | [55.8 to 77.2] | 2.8 | [-5.5 to 11.1] | .506 |
| *Role-Physical* | | |  |  |  |  |  | |  | |  | |  |  |  |  |  |
|  | Baseline | |  |  |  |  |  | |  | |  | |  |  |  |  |  |
|  |  | Elementary/high school | 31 | 13.7 | [2.1 to 25.3] |  |  | |  | | 49 | | 15.8 | [6.4 to 25.2] |  |  |  |
|  |  | University/college ≤ 4 years | 28 | 25.9 | [13.7 to 38.1] |  |  | |  | | 55 | | 23.6 | [14.8 to 32.5] |  |  |  |
|  |  | University/college > 4 years | 14 | 14.3 | [-3.0 to 31.6] |  |  | |  | | 21 | | 17.9 | [3.5 to 32.2] |  |  |  |
|  | 3 months | |  |  |  |  |  | |  | |  | |  |  |  |  |  |
|  |  | Elementary/high school | 31 | 17.5 | [3.7 to 31.2] |  |  | |  | | 49 | | 21.6 | [11.6 to 31.6] |  |  |  |
|  |  | University/college ≤ 4 years | 28 | 27.2 | [13.8 to 40.6] | -2.5 | [-20.3 to 15.4] | | .786 | | 55 | | 26.3 | [16.9 to 35.6] | -3.1 | [-17.5 to 11.2] | .666 |
|  |  | University/college > 4 years | 14 | 44.2 | [24.3 to 64.1] | 26.2 | [3.6 to 48.7] | | **.023** | | 21 | | 31.5 | [16.6 to 46.4] | 7.9 | [-10.9 to 26.7] | .413 |
|  | 6 months | |  |  |  |  |  | |  | |  | |  |  |  |  |  |
|  |  | Elementary/high school | 31 | 19.8 | [5.2 to 34.4] |  |  | |  | | 49 | | 22.5 | [11.9 to 33.0] |  |  |  |
|  |  | University/college ≤ 4 years | 28 | 37.1 | [23.0 to 51.1] | 5.0 | [-13.9 to 24.0] | | .602 | | 55 | | 27.3 | [17.7 to 37.0] | -3.0 | [-17.9 to 11.9] | .696 |
|  |  | University/college > 4 years | 14 | 38.2 | [18.1 to 58.2] | 17.7 | [-5.3 to 40.9] | | .132 | | 21 | | 30.0 | [14.8 to 45.3] | 5.5 | [-13.8 to 24.9] | .575 |
|  | 12 months | |  |  |  |  |  | |  | |  | |  |  |  |  |  |
|  |  | Elementary/high school | 31 | 13.7 | [-3.5 to 30.9] |  |  | |  | | 49 | | 20.3 | [9.1 to 30.7] |  |  |  |
|  |  | University/college ≤ 4 years | 28 | 35.2 | [21.2 to 49.3] | 9.3 | [-11.7 to 30.3] | | .383 | | 55 | | 28.2 | [18.7 to 37.8] | 0.1 | [-14.6 to 14.8] | .991 |
|  |  | University/college > 4 years | 14 | 29.8 | [9.8 to 49.8] | 15.1 | [-9.3 to 40.4] | | .220 | | 21 | | 35.3 | [19.4 to 51.2] | 13.0 | [-6.8 to 32.7] | .199 |
| *Bodily pain* | | |  |  |  |  |  | |  | |  | |  |  |  |  |  |
|  | Baseline | |  |  |  |  |  | |  | |  | |  |  |  |  |  |
|  |  | Elementary/high school | 31 | 21.0 | [14.8 to 27.3] |  |  | |  | | 49 | | 24.5 | [19.6 to 29.5] |  |  |  |
|  |  | University/college ≤ 4 years | 28 | 28.5 | [22.0 to 35.0] |  |  | |  | | 55 | | 28.4 | [23.7 to 33.0] |  |  |  |
|  |  | University/college > 4 years | 14 | 33.2 | [24.0 to 42.4] |  |  | |  | | 21 | | 31.9 | [24.3 to 39.4] |  |  |  |
|  | 3 months | |  |  |  |  |  | |  | |  | |  |  |  |  |  |
|  |  | Elementary/high school | 31 | 25.3 | [17.8 to 32.8] |  |  | |  | | 49 | | 24.3 | [19.1 to 29.6] |  |  |  |
|  |  | University/college ≤ 4 years | 28 | 31.8 | [24.6 to 39.1] | -0.9 | [-11.1 to 9.3] | | .863 | | 55 | | 32.6 | [27.7 to 37.5] | 4.4 | [-2.6 to 11.5] | .214 |
|  |  | University/college > 4 years | 14 | 46.2 | [35.4 to 57.0] | 8.7 | [-4.1 to 21.6] | | .185 | | 21 | | 40.3 | [32.5 to 48.2] | 8.6 | [-0.6 to 17.8] | .066 |
|  | 6 months | |  |  |  |  |  | |  | |  | |  |  |  |  |  |
|  |  | Elementary/high school | 31 | 26.0 | [18.0 to 33.9] |  |  | |  | | 49 | | 27.6 | [22.1 to 33.2] |  |  |  |
|  |  | University/college ≤ 4 years | 28 | 39.8 | [32.2 to 47.5] | 6.4 | [-4,4 to 17.2] | | .246 | | 55 | | 36.4 | [31.4 to 41.5] | 5.0 | [-2.4 to 12.3] | .184 |
|  |  | University/college > 4 years | 14 | 45.3 | [34.5 to 56.2] | 7.2 | [-6.0 to 20.4] | | .288 | | 21 | | 35.1 | [27.2 to 43.2] | 0.2 | [-9.3 to 9.7] | 1.0 |
|  | 12 months | |  |  |  |  |  | |  | |  | |  |  |  |  |  |
|  |  | Elementary/high school | 31 | 21.5 | [12.0 to 31.0] |  |  | |  | | 49 | | 29.7 | [24.3 to 35.2] |  |  |  |
|  |  | University/college ≤ 4 years | 28 | 40.3 | [32.7 to 47.9] | 11.4 | [-0.6 to 23.4] | | .063 | | 55 | | 38.0 | [33.0 to 43.0] | 4.4 | [-2.8 to 11.6] | .230 |
|  |  | University/college > 4 years | 14 | 40.0 | [29.2 to 50.9] | 6.4 | [-7.8 to 20.6] | | .375 | | 21 | | 39.3 | [31.0 to 47.6] | 2.3 | [-7.5 to 12.0] | .649 |
| General health | | |  |  |  |  |  | |  | |  | |  |  |  |  |  |
|  | Baseline | |  |  |  |  |  | |  | |  | |  |  |  |  |  |
|  |  | Elementary/high school | 31 | 30.6 | [24.5 to 36.8] |  |  | |  | | 49 | | 34.3 | [28.9 to 39.7] |  |  |  |
|  |  | University/college ≤ 4 years | 28 | 40.2 | [33.7 to 46.7] |  |  | |  | | 55 | | 37.2 | [32.1 to 42.3] |  |  |  |
|  |  | University/college > 4 years | 14 | 48.2 | [39.0 to 57.4] |  |  | |  | | 21 | | 43.3 | [35.1 to 51.6] |  |  |  |
|  | 3 months | |  |  |  |  |  | |  | |  | |  |  |  |  |  |
|  |  | Elementary/high school | 31 | 27.7 | [20.5 to 35.0] |  |  | |  | | 49 | | 39.3 | [33.6 to 44.9] |  |  |  |
|  |  | University/college ≤ 4 years | 28 | 41.4 | [34.3 to 48.4] | 4.1 | [-4.9 to 13.2] | | .371 | | 55 | | 42.4 | [37.1 to 47.7] | 0.3 | [-5.9 to 6.4] | .935 |
|  |  | University/college > 4 years | 14 | 55.3 | [44.8 to 65.8] | 10.1 | [-1.4 to 21.5] | | .084 | | 21 | | 47.3 | [38.9 to 55.8] | -1.0 | [-9.1 to 7.1] | .810 |
|  | 6 months | |  |  |  |  |  | |  | |  | |  |  |  |  |  |
|  |  | Elementary/high school | 31 | 37.5 | [29.9 to 45.2] |  |  | |  | | 49 | | 37.1 | [31.3 to 43.0] |  |  |  |
|  |  | University/college ≤ 4 years | 28 | 44.7 | [37.3 to 52.1] | -2.4 | [-12.0 to 7.2] | | .628 | | 55 | | 42.4 | [37.0 to 47.7] | 2.3 | [-4.1 to 8.8] | .476 |
|  |  | University/college > 4 years | 14 | 60.1 | [49.6 to 70.7] | 5.0 | [-6.7 to 16.8] | | .400 | | 21 | | 45.8 | [37.2 to 54.3] | -0.4 | [-8.7 to 7.9] | .926 |
|  | 12 months | |  |  |  |  |  | |  | |  | |  |  |  |  |  |
|  |  | Elementary/high school | 31 | 31.2 | [22.3 to 40.1] |  |  | |  | | 49 | | 37.8 | [32.0 to 43.5] |  |  |  |
|  |  | University/college ≤ 4 years | 28 | 42.0 | [34.6 to 49.4] | 1.3 | [-9.4 to 11.9] | | .814 | | 55 | | 41.4 | [36.0 to 46.7] | 0.7 | [-5.6 to 7.1] | .822 |
|  |  | University/college > 4 years | 14 | 57.9 | [47.4 to 68.5] | 9.1 | [-3.4 to 21.7] | | .155 | | 21 | | 50.9 | [42.2 to 59.7] | 4.1 | [-4.4 to 12.6] | .342 |
| *Vitality* | | |  |  |  |  |  | |  | |  | |  |  |  |  |  |
|  | Baseline | |  |  |  |  |  | |  | |  | |  |  |  |  |  |
|  |  | Elementary/high school | 31 | 20.5 | [13.8 to 32.0] |  |  | |  | | 49 | | 25.8 | [20.1 to 31.6] |  |  |  |
|  |  | University/college ≤ 4 years | 28 | 28.4 | [21.3 to 35.5] |  |  | |  | | 55 | | 26.8 | [21.4 to 32.2] |  |  |  |
|  |  | University/college > 4 years | 14 | 25.0 | [15.0 to 35.0] |  |  | |  | | 21 | | 29.8 | [21.0 to 38.5] |  |  |  |
|  | 3 months | |  |  |  |  |  | |  | |  | |  |  |  |  |  |
|  |  | Elementary/high school | 31 | 24.1 | [16.2 to 32.0] |  |  | |  | | 49 | | 27.7 | [21.8 to 33.7] |  |  |  |
|  |  | University/college ≤ 4 years | 28 | 30.2 | [22.5 to 37.9] | -1.8 | [-11.8 to 8.2] | | .723 | | 55 | | 36.8 | [31.2 to 42.4] | 8.1 | [1.6 to 14.6] | .015 |
|  |  | University/college > 4 years | 14 | 44.4 | [32.9 to 55.8] | 15.7 | [3.1 to 28.4] | | **.015** | | 21 | | 38.2 | [29.3 to 47.2] | 6.5 | [-2.0 to 15.1] | .131 |
|  | 6 months | |  |  |  |  |  | |  | |  | |  |  |  |  |  |
|  |  | Elementary/high school | 31 | 24.4 | [16.0 to 32.8] |  |  | |  | | 49 | | 27.0 | [20.8 to 33.2] |  |  |  |
|  |  | University/college ≤ 4 years | 28 | 31.3 | [23.2 to 39.4] | -1.0 | [-11.6 to 9.6] | | .854 | | 55 | | 28.9 | [23.2 to 34.6] | 0.9 | [-5.8 to 7.7] | .786 |
|  |  | University/college > 4 years | 14 | 43.1 | [31.6 to 54.6] | 14.2 | [1.2 to 27.1] | | **.033** | | 21 | | 38.7 | [29.6 to 47.8] | 7.8 | [-1.0 to 16.6] | .084 |
|  | 12 months | |  |  |  |  |  | |  | |  | |  |  |  |  |  |
|  |  | Elementary/high school | 31 | 19.5 | [9.7 to 29.2] |  |  | |  | | 49 | | 30.9 | [24.8 to 37.0] |  |  |  |
|  |  | University/college ≤ 4 years | 28 | 25.7 | [17.6 to 33.8] | -1.6 | [-13.4 to 10.1] | | .784 | | 55 | | 32.9 | [27.2 to 38.5] | 0.9 | [-5.8 to 7.6] | .784 |
|  |  | University/college > 4 years | 14 | 34.7 | [23.2 to.46.2] | 10.8 | [-3.2 to 24.7] | | .131 | | 21 | | 38.2 | [28.9 to 47.5] | 3.4 | [-5.6 to 12.4] | .464 |
| *Social functioning* | | |  |  |  |  |  | |  | |  | |  |  |  |  |  |
|  | Baseline | |  |  |  |  |  | |  | |  | |  |  |  |  |  |
|  |  | Elementary/high school | 31 | 38.3 | [29.7 to 46.9] |  |  | |  | | 49 | | 42.1 | [34.9 to 49.2] |  |  |  |
|  |  | University/college ≤ 4 years | 28 | 51.3 | [42.3 to 60.4] |  |  | |  | | 55 | | 50.0 | [43.3 to 56.7] |  |  |  |
|  |  | University/college > 4 years | 14 | 49.1 | [36.3 to 61.9] |  |  | |  | | 21 | | 48.8 | [37.9 to 59.7] |  |  |  |
|  | 3 months | |  |  |  |  |  | |  | |  | |  |  |  |  |  |
|  |  | Elementary/high school | 31 | 45.7 | [35.7 to 55.8] |  |  | |  | | 49 | | 51.8 | [44.3 to 59.3] |  |  |  |
|  |  | University/college ≤ 4 years | 28 | 47.5 | [37.7 to 57.4] | -11.2 | [-23.8 to 1.3] | | .080 | | 55 | | 53.0 | [45.9 to 60.0] | -6.7 | [-16.3 to 2.8] | .167 |
|  |  | University/college > 4 years | 14 | 58.7 | [44.1 to 73.3] | 2.1 | [-13.8 to 18.0] | | .794 | | 21 | | 60.7 | [49.5 to 72.0] | 2.2 | [-10.4 to 14.8] | .732 |
|  | 6 months | |  |  |  |  |  | |  | |  | |  |  |  |  |  |
|  |  | Elementary/high school | 31 | 51.8 | [41.1 to 62.4] |  |  | |  | | 49 | | 54.9 | [47.0 to 62.8] |  |  |  |
|  |  | University/college ≤ 4 years | 28 | 51.9 | [41.7 to 62.2] | -12.8 | [-26.2 to 0.5] | | .060 | | 55 | | 57.6 | [50.4 to 64.8] | -5.2 | [-15.2 to 4.8] | .307 |
|  |  | University/college > 4 years | 14 | 60.4 | [45.7 to 75.0] | -2.2 | [-18.5 to 14.1] | | .793 | | 21 | | 56.1 | [44.7 to 67.5] | -5.5 | [-18.4 to 7.4] | .405 |
|  | 12 months | |  |  |  |  |  | |  | |  | |  |  |  |  |  |
|  |  | Elementary/high school | 31 | 42.1 | [29.7 to 54.5] |  |  | |  | | 49 | | 53.0 | [45.2 to 60.7] |  |  |  |
|  |  | University/college ≤ 4 years | 28 | 53.9 | [43.6 to 64.2] | -1.2 | [-16.1 to 13.6] | | .869 | | 55 | | 56.2 | [49.0 to 63.3] | -4.7 | [-14.6 to 5.1] | .347 |
|  |  | University/college > 4 years | 14 | 67.3 | [52.7 to 82.0] | 14 | [-3.1 to 32.0] | | .107 | | 21 | | 60.6 | [48.8 to 72.5] | 0.9 | [-12.3 to 14.1] | .892 |
| *Role-Emotional* | | |  |  |  |  |  | |  | |  | |  |  |  |  |  |
|  | Baseline | |  |  |  |  |  | |  | |  | |  |  |  |  |  |
|  |  | Elementary/high school | 31 | 40.9 | [26.8 to 60.0] |  |  | |  | | 49 | | 43.5 | [31.3 to 55.7] |  |  |  |
|  |  | University/college ≤ 4 years | 28 | 59.5 | [44.7 to 74.4] |  |  | |  | | 55 | | 52.1 | [40.6 to 63.6] |  |  |  |
|  |  | University/college > 4 years | 14 | 69.0 | [48.1 to 90.0] |  |  | |  | | 21 | | 50.8 | [32.2 to 69.4] |  |  |  |
|  | 3 months | |  |  |  |  |  | |  | |  | |  |  |  |  |  |
|  |  | Elementary/high school | 31 | 52.0 | [34.2 to 69.8] |  |  | |  | | 49 | | 49.8 | [36.7 to 62.8] |  |  |  |
|  |  | University/college ≤ 4 years | 28 | 55.3 | [38.4 to 72.2] | -15.4 | [-43.8 to 13.0] | | .289 | | 55 | | 59.6 | [47.4 to 71.8] | 1.3 | [-18.9 to 21.4] | .901 |
|  |  | University/college > 4 years | 14 | 68.2 | [42.5 to 93.9] | -12.0 | [-47.9 to 23.9] | | .514 | | 21 | | 71.3 | [51.9 to 90.7] | 14.3 | [-12.2 to 40.8] | .290 |
|  | 6 months | |  |  |  |  |  | |  | |  | |  |  |  |  |  |
|  |  | Elementary/high school | 31 | 33.9 | [14.6 to 53.1] |  |  | |  | | 49 | | 55.7 | [41.8 to 69.6] |  |  |  |
|  |  | University/college ≤ 4 years | 28 | 54.3 | [36.1 to 72.5] | 1.8 | [-28.3 to 31.8] | | .908 | | 55 | | 47.0 | [34.3 to 59.6] | -17.3 | [-38.3 to 3.7] | .106 |
|  |  | University/college > 4 years | 14 | 78.6 | [52.9 to 104.4] | 16.6 | [-20.1 to 53.3] | | .375 | | 21 | | 60.5 | [40.6 to 80.3] | -2.5 | [-29.8 to 24.8] | .859 |
|  | 12 months | |  |  |  |  |  | |  | |  | |  |  |  |  |  |
|  |  | Elementary/high school | 31 | 31.1 | [7.2 to 55.0] |  |  | |  | | 49 | | 52.4 | [38.8 to 66.0] |  |  |  |
|  |  | University/college ≤ 4 years | 28 | 52.6 | [34.5 to 70.8] | 2.8 | [30.4 to 36.1] | | .868 | | 55 | | 66.5 | [54.0 to 79.0] | 5.5 | [-15.2 to 26.2] | .603 |
|  |  | University/college > 4 years | 14 | 78.6 | [52.9 to 104.4] | 19.3 | [20.0 to 58.7] | | .336 | | 21 | | 73.6 | [52.7 to 94.5] | 14.0 | [-13.9 to 41.8] | .325 |
| *Mental health* | | |  |  |  |  |  | |  | |  | |  |  |  |  |  |
|  | Baseline | |  |  |  |  |  | |  | |  | |  |  |  |  |  |
|  |  | Elementary/high school | 31 | 61.8 | [56.1 to 67.6] |  |  | |  | | 49 | | 62.9 | [57.7 to 68.1] |  |  |  |
|  |  | University/college ≤ 4 years | 28 | 62.4 | [56.4 to 68.5] |  |  | |  | | 55 | | 66.7 | [61.8 to 71.6] |  |  |  |
|  |  | University/college > 4 years | 14 | 64.0 | [55.5 to 72.5] |  |  | |  | | 21 | | 70.7 | [62.7 to 78.6] |  |  |  |
|  | 3 months | |  |  |  |  |  | |  | |  | |  |  |  |  |  |
|  |  | Elementary/high school | 31 | 61.1 | [54.1 to 68.4] |  |  | |  | | 49 | | 63.7 | [58.3 to 69.1] |  |  |  |
|  |  | University/college ≤ 4 years | 28 | 61.7 | [54.9 to 68.4] | 0,0 | [-9,7 to 9,7] | | .998 | | 55 | | 70.5 | [65.4 to 75.6] | 3.1 | [-2.9 to 9.0] | .314 |
|  |  | University/college > 4 years | 14 | 73.9 | [63.8 to 84.0] | 10.6 | [-1.6 to 22.9] | | .089 | | 21 | | 73.3 | [65.2 to 81.4] | 1.9 | [-5.9 to 9.7] | .638 |
|  | 6 months | |  |  |  |  |  | |  | |  | |  |  |  |  |  |
|  |  | Elementary/high school | 31 | 62.2 | [54.8 to 69.6] |  |  | |  | | 49 | | 63.8 | [58.1 to 69.4] |  |  |  |
|  |  | University/college ≤ 4 years | 28 | 62.1 | [55.0 to 69.2] | -0.7 | [-11.0 to 9.5] | | .888 | | 55 | | 66.5 | [61.3 to 71.7] | -1.0 | [-7.2 to 5.2] | .752 |
|  |  | University/college > 4 years | 14 | 75.0 | [64.9 to 85.1] | 10.6 | [-2.0 to 23.1] | | .099 | | 21 | | 67.4 | [59.1 to 75.6] | -4.1 | [-12.2 to 4.0] | .320 |
|  | 12 months | |  |  |  |  |  | |  | |  | |  |  |  |  |  |
|  |  | Elementary/high school | 31 | 56.8 | [47.9 to 65.7] |  |  | |  | | 49 | | 64.0 | [58.4 to 69.5] |  |  |  |
|  |  | University/college ≤ 4 years | 28 | 63.6 | [56.5 to 70.7] | 6.2 | [-5.2 to 17.6] | | .288 | | 55 | | 69.6 | [64.4 to 74.7] | 1.9 | [-4.3 to 8.0] | .550 |
|  |  | University/college > 4 years | 14 | 72.7 | [62.7 to 82.9] | 13.8 | [0.3 to 27.3] | | **.045** | | 21 | | 72,8 | [64.3 to 81.3] | 1.1 | [-7.1 to 9.4] | .711 |
| Pain catastrophizing (PCS^i^) | | |  |  |  |  |  | |  | |  | |  |  |  |  |  |
| *Rumination* | | |  |  |  |  |  | |  | |  | |  |  |  |  |  |
|  | Baseline | |  |  |  |  |  | |  | |  | |  |  |  |  |  |
|  |  | Elementary/high school | 31 | 8.4 | [7.0 to 9.8] |  |  | |  | | 49 | | 8.0 | [7.0 to 9.1] |  |  |  |
|  |  | University/college ≤ 4 years | 28 | 7.3 | [5.8 to 8.8] |  |  | |  | | 55 | | 7.3 | [6.4 to 8.3] |  |  |  |
|  |  | University/college > 4 years | 14 | 6.6 | [4.6 to 8.7] |  |  | |  | | 21 | | 5.0 | [3.4 to 6.6] |  |  |  |
|  | 3 months | |  |  |  |  |  | |  | |  | |  |  |  |  |  |
|  |  | Elementary/high school | 31 | 8.6 | [7.0 to 10.2] |  |  | |  | | 49 | | 7.3 | [6.2 to 8.4] |  |  |  |
|  |  | University/college ≤ 4 years | 28 | 5.5 | [3.9 to 7.1] | -2.0 | [-4.0 to 0.1] | | .060 | | 55 | | 6.3 | [5.2 to 7.3] | -0.4 | [-1.7 to 1.0] | .600 |
|  |  | University/college > 4 years | 14 | 5.3 | [2.9 to 7.6] | -1.6 | [-4.2 to 1.0] | | .230 | | 21 | | 4.7 | [3.0 to 6.3] | 0.4 | [-1.3 to 2.1] | .667 |
|  | 6 months | |  |  |  |  |  | |  | |  | |  |  |  |  |  |
|  |  | Elementary/high school | 31 | 8.7 | [7.0 to 10.4] |  |  | |  | | 49 | | 6.6 | [5.5 to 7.6] |  |  |  |
|  |  | University/college ≤ 4 years | 28 | 6.0 | [4.4 to 7.7] | -1.6 | [-3.7 to 0.6] | | .162 | | 55 | | 6.1 | [5.1 to 7.2] | 0.2 | [-1.2 to 1.6] | .752 |
|  |  | University/college > 4 years | 14 | 3.9 | [1.5 to 6.3] | -3.0 | [-5.7 to -0.4] | | **.027** | | 21 | | 4.2 | [2.6 to 5.8] | 0.6 | [-1.2 to 2.4] | .521 |
|  | 12 months | |  |  |  |  |  | |  | |  | |  |  |  |  |  |
|  |  | Elementary/high school | 31 | 8.3 | [6.3 to 10.3] |  |  | |  | | 49 | | 6.2 | [5.1 to 7.3] |  |  |  |
|  |  | University/college ≤ 4 years | 28 | 6.1 | [4.4 to 7.8] | -1.1 | [-3.5 to 1.3] | | .369 | | 55 | | 5.1 | [4.0 to 6.1] | -0.5 | [-1.8 to 0.9] | .511 |
|  |  | University/college > 4 years | 14 | 3.1 | [0.8 to 5.5] | -3.4 | [-6.3 to -0.6] | | **.019** | | 21 | | 3.9 | [2.2 to 5.6] | 0.7 | [-1.1 to 2.5] | .447 |
| *Magnification* | | |  |  |  |  |  | |  | |  | |  |  |  |  |  |
|  | Baseline | |  |  |  |  |  | |  | |  | |  |  |  |  |  |
|  |  | Elementary/high school | 31 | 4.1 | [3.4 to 4.9] |  |  | |  | | 49 | | 3.9 | [3.3 to 4.6] |  |  |  |
|  |  | University/college ≤ 4 years | 28 | 3.6 | [2.8 to 4.5] |  |  | |  | | 55 | | 3.0 | [2.4 to 3.6] |  |  |  |
|  |  | University/college > 4 years | 14 | 3.2 | [2.1 to 4.4] |  |  | |  | | 21 | | 2.5 | [1.6 to 3.5] |  |  |  |
|  | 3 months | |  |  |  |  |  | |  | |  | |  |  |  |  |  |
|  |  | Elementary/high school | 31 | 4.1 | [3.1 to 5.0] |  |  | |  | | 49 | | 3.6 | [2.9 to 4.3] |  |  |  |
|  |  | University/college ≤ 4 years | 28 | 2.8 | [1.9 to 3.7] | -0.8 | [-2.2 to 0,6] | | .248 | | 55 | | 2.7 | [2.1 to 3.3] | 0.0 | [-0.8 to 0.8] | .943 |
|  |  | University/college > 4 years | 14 | 2.7 | [1.3 to 4.0] | -0,5 | [-2.2 to 1.2] | | .574 | | 21 | | 2.3 | [1.3 to 3.3] | 0.1 | [-1.0 to 1.1] | .899 |
|  | 6 months | |  |  |  |  |  | |  | |  | |  |  |  |  |  |
|  |  | Elementary/high school | 31 | 4.2 | [3.2 to 5.2] |  |  | |  | | 49 | | 3.5 | [2.8 to 4.2] |  |  |  |
|  |  | University/college ≤ 4 years | 28 | 3.4 | [2.4 to 4.4] | -0.3 | [-1.8 to 1.1] | | .656 | | 55 | | 2.6 | [1.9 to 3.2] | 0.0 | [-0.8 to 0.8] | .944 |
|  |  | University/college > 4 years | 14 | 2.1 | [0.8 to 3.5] | -1.2 | [-2.9 to 0.6] | | .190 | | 21 | | 2.2 | [1.1 to 3.2] | 0.0 | [-1.1 to 1.1] | .931 |
|  | 12 months | |  |  |  |  |  | |  | |  | |  |  |  |  |  |
|  |  | Elementary/high school | 31 | 3.5 | [2.3 to 4.7] |  |  | |  | | 49 | | 3.3 | [2.7 to 4.0] |  |  |  |
|  |  | University/college ≤ 4 years | 28 | 3.1 | [2.1 to 4.0] | 0.1 | [-1.5 to 1.7] | | .926 | | 55 | | 2.1 | [1.4 to 2.7] | -0.4 | [-1.2 to 0.4] | .322 |
|  |  | University/college > 4 years | 14 | 1.8 | [0.4 to 3.2] | -0.8 | [-2.7 to 1.1] | | .420 | | 21 | | 1.9 | [0.9 to 3.0] | 0.0 | [-1.1 to 1.1] | .956 |
| *Helplessness* | | |  |  |  |  |  | |  | |  | |  |  |  |  |  |
|  | Baseline | |  |  |  |  |  | |  | |  | |  |  |  |  |  |
|  |  | Elementary/high school | 31 | 10.8 | [9.1 to 12.6] |  |  | |  | | 49 | | 9.7 | [8.4 to 11.0] |  |  |  |
|  |  | University/college ≤ 4 years | 28 | 9.8 | [7.9 to 11.7] |  |  | |  | | 55 | | 7.9 | [6.6 to 9.1] |  |  |  |
|  |  | University/college > 4 years | 14 | 7.9 | [5.2 to 10.5] |  |  | |  | | 21 | | 5.6 | [3.6 to 7.5] |  |  |  |
|  | 3 months | |  |  |  |  |  | |  | |  | |  |  |  |  |  |
|  |  | Elementary/high school | 31 | 11.3 | [9.2 to 13.4] |  |  | |  | | 49 | | 8.7 | [7.4 to 10.0] |  |  |  |
|  |  | University/college ≤ 4 years | 28 | 7.6 | [5.5 to 9.6] | -2.7 | [-5.5 to 0.1] | | .061 | | 55 | | 7.4 | [6.2 to 8.6] | 0.5 | [-1.0 to 2.0] | .493 |
|  |  | University/college > 4 years | 14 | 5.1 | [2.0 to 8.1] | -3.2 | [-6.8 to 0.3] | | .074 | | 21 | | 6.6 | [4.6 to 8.6] | 2.0 | [0.1 to 4.0] | .044 |
|  | 6 months | |  |  |  |  |  | |  | |  | |  |  |  |  |  |
|  |  | Elementary/high school | 31 | 9.9 | [7.6 to 12.1] |  |  | |  | | 49 | | 8.9 | [7.5 to 10.3] |  |  |  |
|  |  | University/college ≤ 4 years | 28 | 6.9 | [4.7 to 9.0] | -2.0 | [-4.9 to 1.0] | | .198 | | 55 | | 7.0 | [5.7 to 8.3] | 0.0 | [-1.6 to 1.5] | .921 |
|  |  | University/college > 4 years | 14 | 4.1 | [1.0 to 7.2] | -2.8 | [-6.5 to 0.8] | | .127 | | 21 | | 4.8 | [2.8 to 6.8] | 0.0 | [-2.0 to 2.0] | .985 |
|  | 12 months | |  |  |  |  |  | |  | |  | |  |  |  |  |  |
|  |  | Elementary/high school | 31 | 10.3 | [7.7 to 13.0] |  |  | |  | | 49 | | 7.8 | [6.5 to 9.2] |  |  |  |
|  |  | University/college ≤ 4 years | 28 | 8.8 | [6.7 to 11.0] | -0.4 | [-3.7 to 2.9] | | .799 | | 55 | | 6.3 | [5.0 to 7.6] | 0.3 | [-1.3 to 1.9] | .713 |
|  |  | University/college > 4 years | 14 | 4.0 | [0.9 to 7.0] | -3.4 | [-7.3 to 0.5] | | .090 | | 21 | | 4.7 | [2.6 to 6.8] | 1.0 | [-1.1 to 3.0] | .367 |
| *PCS total* | | |  |  |  |  |  | |  | |  | |  |  |  |  |  |
|  | Baseline | |  |  |  |  |  | |  | |  | |  |  |  |  |  |
|  |  | Elementary/high school | 31 | 23.4 | [19.7 to 26.8] |  |  | |  | | 49 | | 21.6 | [19.0 to 24.2] |  |  |  |
|  |  | University/college ≤ 4 years | 28 | 20.7 | [17.0 to 24.4] |  |  | |  | | 55 | | 18.2 | [15.8 to 20.7] |  |  |  |
|  |  | University/college > 4 years | 14 | 17.7 | [12.5 to 23.0] |  |  | |  | | 21 | | 13.1 | [9.1 to 17.1] |  |  |  |
|  | 3 months | |  |  |  |  |  | |  | |  | |  |  |  |  |  |
|  |  | Elementary/high school | 31 | 23.9 | [19.8 to 28.0] |  |  | |  | | 49 | | 19.6 | [16.9 to 22.3] |  |  |  |
|  |  | University/college ≤ 4 years | 28 | 15.9 | [11.9 to 19.9] | -5.4 | [-10.5 to -0.3] | | **.039** | | 55 | | 16.4 | [13.8 to 18.9] | 0.1 | [-2.8 to 3.0] | .922 |
|  |  | University/college > 4 years | 14 | 12.9 | [7.0 to 18.8] | -5.3 | [-11.8 to 1.1] | | .105 | | 21 | | 13.6 | [9.5 to 17.6] | 2.5 | [-1.3 to 6.3] | .202 |
|  | 6 months | |  |  |  |  |  | |  | |  | |  |  |  |  |  |
|  |  | Elementary/high school | 31 | 22.8 | [18.4 to 27.1] |  |  | |  | | 49 | | 19.0 | [16.2 to 21.8] |  |  |  |
|  |  | University/college ≤ 4 years | 28 | 16.2 | [12.1 to 20.4] | -3.9 | [-9.3 to 1.5] | | .159 | | 55 | | 15.7 | [13.1 to 18.3] | 0.1 | [-2.9 to 3.2] | .928 |
|  |  | University/college > 4 years | 14 | 10.0 | [4.0 to 15.9] | -7.2 | [-13.8 to -0.5] | | **.034** | | 21 | | 11.1 | [7.0 to 15.2] | 0.7 | [-3.2 to 4.6] | .735 |
|  | 12 months | |  |  |  |  |  | |  | |  | |  |  |  |  |  |
|  |  | Elementary/high school | 31 | 22.0 | [17.0 to 27.0] |  |  | |  | | 49 | | 17.4 | [14.7 to 20.2] |  |  |  |
|  |  | University/college ≤ 4 years | 28 | 18.0 | [13.8 to 22.1] | -1.3 | [-7.4 to 4.6] | | .656 | | 55 | | 13.5 | [10.9 to 16.0] | -0.5 | [-3.5 to 2.4] | .719 |
|  |  | University/college > 4 years | 14 | 8.7 | [2.8 to 14.7] | -7.6 | [-14.7 to -0.5] | | **.035** | | 21 | | 10.5 | [6.3 to 14.7] | 1.6 | [-2.4 to 5.6] | .437 |
| Chronic pain acceptance (CPAQ^j^) | | |  |  |  |  |  | |  | |  | |  |  |  |  |  |
| *Willingness* | | |  |  |  |  |  | |  | |  | |  |  |  |  |  |
|  | Baseline | |  |  |  |  |  | |  | |  | |  |  |  |  |  |
|  |  | Elementary/high school | 31 | 12.9 | [12.0 to 13.8] |  |  | |  | | 49 | | 13.0 | [12.2 to 13.8] |  |  |  |
|  |  | University/college ≤ 4 years | 28 | 13.5 | [12.6 to 14.4] |  |  | |  | | 55 | | 13.9 | [13.2 to 14.7] |  |  |  |
|  |  | University/college > 4 years | 14 | 14.3 | [13.0 to 15.6] |  |  | |  | | 21 | | 13.9 | [12.7 to 15.1] |  |  |  |
|  | 3 months | |  |  |  |  |  | |  | |  | |  |  |  |  |  |
|  |  | Elementary/high school | 31 | 14.2 | [13.1 to 15.3] |  |  | |  | | 49 | | 13.7 | [12.8 to 14.5] |  |  |  |
|  |  | University/college ≤ 4 years | 28 | 14.0 | [13.0 to 15.0] | -0.8 | [-2.6 to 1.0] | | .368 | | 55 | | 13.9 | [13.1 to 14.7] | -0.7 | [-1.9 to 0.6] | .287 |
|  |  | University/college > 4 years | 14 | 14.3 | [12.7 to 15.9] | -1.3 | [-3.5 to 0.9] | | .245 | | 21 | | 13.5 | [12.3 to 14.8] | -1.0 | [-2.7 to 0.6] | .227 |
|  | 6 months | |  |  |  |  |  | |  | |  | |  |  |  |  |  |
|  |  | Elementary/high school | 31 | 13.6 | [12.4 to 14.8] |  |  | |  | | 49 | | 13.5 | [12.6 to 14.4] |  |  |  |
|  |  | University/college ≤ 4 years | 28 | 14.2 | [13.0 to 15.2] | -0.0 | [-1.9 to 1.8] | | **.**976 | | 55 | | 13.8 | [13.0 to 14.6] | -0.6 | [-1.9 to 0.7] | .349 |
|  |  | University/college > 4 years | 14 | 15.6 | [14.0 to 17.1] | 0.6 | [-1.7 to 2.9] | | .614 | | 21 | | 13.1 | [11.9 to 14.4] | -1.3 | [-3.0 to 0.4] | .145 |
|  | 12 months | |  |  |  |  |  | |  | |  | |  |  |  |  |  |
|  |  | Elementary/high school | 31 | 14.5 | [13.0 to 16.0] |  |  | |  | | 49 | | 13.9 | [13.0 to 14.8] |  |  |  |
|  |  | University/college ≤ 4 years | 28 | 14.4 | [13.3 to 15.5] | -0.7 | [-2.8 to 1.4] | | .512 | | 55 | | 13.9 | [13.1 to 14.7] | -0.9 | [-2.2 to 0.4] | .166 |
|  |  | University/college > 4 years | 14 | 15.7 | [14.1 to 17.2] | -0.2 | [-2.6 to 2.2] | | .870 | | 21 | | 13.6 | [12.2 to 15.0] | -1.2 | [-2.9 to 0.6] | .191 |
| *Activity engagement* | | |  |  |  |  |  | |  | |  | |  |  |  |  |  |
|  | Baseline | |  |  |  |  |  | |  | |  | |  |  |  |  |  |
|  |  | Elementary/high school | 31 | 12.8 | [11.7 to 14.0] |  |  | |  | | 49 | | 12.8 | [11.9 to 13.8] |  |  |  |
|  |  | University/college ≤ 4 years | 28 | 14.0 | [12.9 to 15.2] |  |  | |  | | 55 | | 13.7 | [12.8 to 14.6] |  |  |  |
|  |  | University/college > 4 years | 14 | 15.1 | [13.5 to 16.8] |  |  | |  | | 21 | | 15.2 | [13.8 to 16.7] |  |  |  |
|  | 3 months | |  |  |  |  |  | |  | |  | |  |  |  |  |  |
|  |  | Elementary/high school | 31 | 13.9 | [12.6 to 15.2] |  |  | |  | | 49 | | 13.6 | [12.6 to 14.6] |  |  |  |
|  |  | University/college ≤ 4 years | 28 | 15.1 | [13.8 to 16.3] | 0.0 | [-1.7 to 1.6] | | .970 | | 55 | | 14.5 | [13.5 to 15.4] | 0.0 | [-1.3 to 1.2] | .966 |
|  |  | University/college > 4 years | 14 | 15.3 | [13.5 to 17.2] | -0.9 | [-2.9 to 1.2] | | .414 | | 21 | | 15.1 | [13.6 to 16.7] | -1.0 | [-2.5 to 1.0] | .321 |
|  | 6 months | |  |  |  |  |  | |  | |  | |  |  |  |  |  |
|  |  | Elementary/high school | 31 | 13.9 | [12.5 to 15.3] |  |  | |  | | 49 | | 13.1 | [12.1 to 14.2] |  |  |  |
|  |  | University/college ≤ 4 years | 28 | 15.3 | [14.0 to 16.6] | 0.2 | [-1.5 to 1.9] | | .829 | | 55 | | 13.9 | [12.9 to 14.9] | -0.1 | [-1.4 to 1.2] | .889 |
|  |  | University/college > 4 years | 14 | 15.9 | [14.0 to 17.8] | -0.3 | [-2.4 to 1.8] | | .765 | | 21 | | 15.1 | [13.5 to 16.6] | -0.4 | [-2.2 to 1.3] | .609 |
|  | 12 months | |  |  |  |  |  | |  | |  | |  |  |  |  |  |
|  |  | Elementary/high school | 31 | 12.8 | [11.2 to 14.4] |  | |  | |  | | 49 | 13.9 | [12.9 to 15.0] |  |  |  |
|  |  | University/college ≤ 4 years | 28 | 14.7 | [13.4 to 16.0] | 0.8 | [-1.2 to 2.7] | | .444 | | 55 | | 14.6 | [13.6 to 15.6] | -0.2 | [-1.5 to 1.0] | .729 |
|  |  | University/college > 4 years | 14 | 14.6 | [12.7 to 16.4] | -0.5 | [-2.8 to 1.8] | | .660 | | 21 | | 15.8 | [14.2 to 17.4] | -0.5 | [-2.3 to 1.2] | .564 |
| *CPAQ total* | | |  |  |  |  |  | |  | |  | |  |  |  |  |  |
|  | Baseline | |  |  |  |  |  | |  | |  | |  |  |  |  |  |
|  |  | Elementary/high school | 31 | 25.7 | [24.2 to 27.3] |  |  | |  | | 49 | | 25.9 | [24.4 to 27.4] |  |  |  |
|  |  | University/college ≤ 4 years | 28 | 27.5 | [25.9 to 29.2] |  |  | |  | | 55 | | 27.7 | [26.2 to 29.1] |  |  |  |
|  |  | University/college > 4 years | 14 | 29.4 | [27.1 to 31.8] |  |  | |  | | 21 | | 29.1 | [26.8 to 31.4] |  |  |  |
|  | 3 months | |  |  |  |  |  | |  | |  | |  |  |  |  |  |
|  |  | Elementary/high school | 31 | 28.2 | [26.3 to 30.6] |  |  | |  | | 49 | | 27.2 | [25.6 to 28.9] |  |  |  |
|  |  | University/college ≤ 4 years | 28 | 29.1 | [27.3 to 31.0] | -0.9 | [-3.7 to 2.0] | | .549 | | 55 | | 28.4 | [26.9 to 29.8] | -0.7 | [-2.8 to 1.4] | .528 |
|  |  | University/college > 4 years | 14 | 29.6 | [26.9 to 32.5] | -2.3 | [-5.9 to 1.3] | | .217 | | 21 | | 28.7 | [26.3 to 31.1] | -1.8 | [-4.6 to 0.9] | .189 |
|  | 6 months | |  |  |  |  |  | |  | |  | |  |  |  |  |  |
|  |  | Elementary/high school | 31 | 27.6 | [25.6 to 29.7] |  |  | |  | | 49 | | 26.6 | [24.9 to 28.3] |  |  |  |
|  |  | University/college ≤ 4 years | 28 | 29.4 | [27.5 to 31.4] | 0.0 | [-3.0 to 3.0] | | .996 | | 55 | | 27.7 | [26.2 to 29.2] | -0.7 | [-2.9 to 1.4] | .516 |
|  |  | University/college > 4 years | 14 | 31.5 | [28.7 to 34.3] | 0.2 | [-3.5 to 3.8] | | .934 | | 21 | | 28.2 | [25.7 to 30.6] | -1.7 | [-4.6 to 1.1] | .231 |
|  | 12 months | |  |  |  |  |  | |  | |  | |  |  |  |  |  |
|  |  | Elementary/high school | 31 | 27.4 | [24.9 to 29.9] |  |  | |  | | 49 | | 27.8 | [26.2 to 29.5] |  |  |  |
|  |  | University/college ≤ 4 years | 28 | 29.1 | [27.1 to 31.0] | -0.1 | [-3.5 to 3.2] | | .938 | | 55 | | 28.5 | [27.0 to 30.0] | -1.1 | [-3.3 to 1.0] | .313 |
|  |  | University/college > 4 years | 14 | 30.3 | [27.4 to 33.1] | -0.8 | [-4.8 to 3.1] | | .684 | | 21 | | 29.4 | [29.9 to 32.0] | -1.6 | [-4.5 to 1.3] | .267 |

^a^BPI: Brief Pain Inventory.

^b^Subscale of the Brief Pain Inventory (score range 0-10; a higher score indicates higher interference in life).

^c^Subscale of the Brief Pain Inventory (score range 0-10; a higher score indicates higher severity).

^d^HADS-A: Hospital Anxiety and Depression Scale-Anxiety subscale (score range 0-21; a higher score indicates a higher degree of anxiety).

^e^HADS-D: Hospital Anxiety and Depression Scale-Depression subscale (score range 0-21; a higher score indicates a higher degree of depression).

^f^SRF-18: Self-regulatory Fatigue 18 scale (score range 18-90; a higher score indicates higher self-regulatory fatigue).

^g^HRQoL: health-related quality of life.

^h^RAND-36: RAND 36-Item scale (score range 0-100; a higher score indicates higher emotional well-being).

^i^PCS: Pain Catastrophizing Scale (score range 0-52; a higher score indicates higher catastrophizing).

^j^CPAQ: Chronic Pain Acceptance Questionnaire (score range 0-52; a higher score indicates a higher acceptance of pain).

Abbreviations: MD, mean difference; CI, confidence interval; β, Standardized Coefficients Beta
