# Supplementary material for: Delivery Model Matters for Digital Interventions in Pain Self-Management: Mixed Methods Study
Source: J Med Internet Res. 2026 Jul 20;28:e89780. doi: 10.2196/89780 (PMC13434256; doi:10.2196/89780)
Supplement: Supplementary file 7 [file jmir_v28i1e89780_app7.docx]

**Multimedia Appendix 7.** Participant education level, program module completion and days used

|  | | EPIO_NoFollowUp_ (n=73^a^) | | | | | EPIO_BlendedCare_ (n=125^b^) | | | | |
| --- | --- | --- | --- | --- | --- | --- | --- | --- | --- | --- | --- |
|  | | **M** | **[95% CI]** | **B** | **[95% CI]** | ***P-value*** | **M** | **[95% CI]** | **B** | **[95% CI]** | *P-value* |
| Program module  completion 3-mos | |  |  |  |  |  |  |  |  |  |  |
|  | Elementary/  high school | 1.7 | [1.3;2.2] |  |  |  | 5.3 | [4.9;5.8] |  |  |  |
|  | University/college  ≤ 4 years | 2.9 | [2.4;3.3] | 1.1 | [0.5;1.8] | **.001** | 5.3 | [4.9;5.7] | -0.1 | [-0.7;0.5] | .860 |
|  | University/college  > 4 years | 3.3 | [2.6;4.0] | 1.5 | [0.7;2.4] | **<.001** | 5.4 | [4.7;6.0] | 0.1 | [-0.7;0.9] | .893 |
| Program module  completion 6-mos | |  |  |  |  |  |  |  |  |  |  |
|  | Elementary/  high school | 2.1 | [1.5;2.6] |  |  |  | 5.9 | [5.4;6.3] |  |  |  |
|  | University/college  ≤ 4 years | 3.3 | [2.7;3.8] | 1.2 | [0.5;2.0] | **.002** | 6.0 | [5.6;6.5] | 0.2 | [-0.4;0.8] | .564 |
|  | University/college  > 4 years | 3.6 | [2.8;4.4] | 1.5 | [0.6;2.5] | **.002** | 6.3 | [5.6;7.0] | 0.4 | [-0.4;1.2] | .300 |
| Program module  completion 12-mos | |  |  |  |  |  |  |  |  |  |  |
|  | Elementary/  high school | 2.6 | [2.0;3.1] |  |  |  | 6.0 | [5.6;6.5] |  |  |  |
|  | University/college  ≤ 4 years | 3.5 | [2.9;4.1] | 0.9 | [0.1;1.7] | **.036** | 6.3 | [6.0;6.8] | 0.3 | [-0.3;1.0] | .279 |
|  | University/college  > 4 years | 3.6 | [2.7;4.4] | 1.0 | [-0.0;2.0] | .057 | 6.8 | [6.1;7.4] | 0.7 | [-0.1;1.5] | .085 |
| Individual days use  of EPIO 3-mos | |  |  |  |  |  |  |  |  |  |  |
|  | Elementary/  high school | 9.3 | [6.8;11.8] |  |  |  | 20.7 | [18.0;23.4] |  |  |  |
|  | University/college  ≤ 4 years | 13.6 | [11.0;16.3] | 4.3 | [0.7;8.0] | **.021** | 27.7 | [25.2;30.2] | 7.0 | [3.3;10.7] | <.001 |
|  | University/college  > 4 years | 19.4 | [15.7;23.2] | 10.1 | [5.6;14.7] | **<.001** | 28.9 | [24.8;33.0] | 8.2 | [3.3;13.1] | .001 |
| Individual days use  of EPIO 6-mos | |  |  |  |  |  |  |  |  |  |  |
|  | Elementary/  high school | 11.8 | [7.9;15.8] |  |  |  | 26.4 | [21.8;31.1] |  |  |  |
|  | University/college  ≤ 4 years | 16.9 | [12.7;21.0] | 5.1 | [-0.6;10.8] | .082 | 37.5 | [33.1;41.9] | 11.0 | [4.6;17.4] | .001 |
|  | University/college  > 4 years | 25.4 | [19.5;31.2] | 13.6 | [6.5;20.6] | **<.001** | 40.8 | [33.7;47.9] | 14.4 | [5.9;22.8] | .001 |
| Individual days use  of EPIO 12-mos | |  |  |  |  |  |  |  |  |  |  |
|  | Elementary/  high school | 16.1 | [10.1;22.2] |  |  |  | 29.9 | [22.8;36.9] |  |  |  |
|  | University/college  ≤ 4 years | 19.9 | [13.5;26.2] | 3.7 | [-5.0;12.5] | .402 | 45.1 | [38.4;51.8] | 15.3 | [5.5;25.0] | .002 |
|  | University/college  > 4 years | 31.2 | [22.2;40.2] | 15.1 | [4.3;25.9] | .006 | 53.1 | [42.3;64.0] | 23.3 | [10.4;36.2] | <.001 |

*Abbreviations: B, standardized coefficients; CI, confidence interval; mos (months)*

*^a^ EPIO_NoFollowUp_: Elementary/high school (n=31), University/college ≤ 4 years (n=28), University/college > 4 years (n=14)*

*^b^ EPIO_BlendedCare_: Elementary/high school (n=49), University/college ≤ 4 years (n=55), University/college > 4 years (n=21)*
